# Allii Macrostemonis Bulbus: A Comprehensive Review of Ethnopharmacology, Phytochemistry and Pharmacology

**DOI:** 10.3390/molecules28062485

**Published:** 2023-03-08

**Authors:** Jianfa Wu, Lulu Wang, Ying Cui, Fei Liu, Jing Zhang

**Affiliations:** 1 Department of Traditional Chinese Medicine, College of Traditional Chinese Medicinal Materials, Jilin Agricultural University, Changchun 130118, China; 2 Department of Traditional Chinese Medicine, College of Medicine, Changchun Sci-Tech University, Changchun 130600, China

**Keywords:** Allii Macrostemonis Bulbus, traditional Chinese medicine, ethnopharmacology, phytochemistry, pharmacological activities, Xiebai

## Abstract

The dried bulbs of Allii Macrostemonis Bulbus (AMB) are called “薤白” in China and are mainly distributed in Asia. The plant species included in the 2020 Edition of the Chinese Pharmacopoeia (ChP) are *Allium macrostemon* Bunge (called xiaogensuan in Chinese, *A. macrostemon*) and *Allium chinense* G. Don (called xie in Chinese, *A. chinense*), respectively. In the traditional Chinese medicine (TCM) theoretical system, AMB is warm in nature, acrid-bitter taste, and attributive to the heart, lung, stomach, large intestine meridian. AMB has the function of activating Yang and removing stasis, regulating Qi and eliminating stagnation. Modern pharmacological studies have shown that AMB has anti-platelet aggregation, hypolipidemic, anti-atherosclerotic, cardiomyocyte, vascular endothelial cell protection, anti-cancer, anti-bacterial, anti-asthmatic, and anti-oxidant effects. In some Asian countries, AMB is often used to treat coronary heart disease (CHD), angina pectoris (AP), asthma, and diarrhea. This review collates the botanical background, ethnopharmacology, phytochemistry, pharmacological activities, quality control, and toxicological studies of AMB, and provides an outlook on the current research deficiencies and future research priorities of AMB, intending to provide ideas for future research directions and commercial development.

## 1. Introduction

AMB is a traditional Chinese herb with homology of medicine and food, named “薤白” in China. The 2020 edition of the ChP includes two basal plants of AMB, *A. macrostemon* and *A. chinense*, with Chinese herb names of “XiaoGenSuan” and “Xie”, respectively [1].

The effect of AMB in traditional Chinese medicine (TCM) is to activate Yang and remove stasis, regulate Qi and eliminate stagnation. It is used to treat chest stuffiness and pains, distention and fullness, stomachache, diarrhea with rectal heaviness, headache, toothache and blood stasis. The mainly components in AMB include steroidal saponins, flavonoids, phenylpropanoids, alkaloids, amino acids, volatile oils, polysaccharides, organic acids and inorganic elements. Modern pharmacological studies show that AMB has effects including anti-platelet aggregation, hypolipidemic, hypoglycemic, antioxidant, cough and asthma, antibacterial, antitumor, antidepressant, etc. [2].

At present, there are many studies on the effects of crude extracts or components of AMB on the treatment of chest pain and diarrhea, but there are few studies on its monomeric activity, quality and safety evaluation, which need to be further studied. Therefore, the literature on AMB should be reviewed and summarized to provide a theoretical basis for further research, expand its application and give full play to its therapeutic effects, so as to better serve human health.

## 2. Method

We conducted literature retrieval of AMB using electronic databases, including PubMed, CNKI, Web of Science, SpecialSciDBS, GBIF, Elsevier, and used national pharmaceutical standards, ancient Chinese medical classics, monographs on TCM, and academic papers to conduct a comprehensive analysis and summary. We used Allii Macrostemonis Bulbus, *Allium macrostemon* Bunge, *Allium chinense* G. Don, phytochemistry, steroidal saponins, pharmacological activity, anti-platelet aggregation, anti-atherosclerosis, cardiomyocytes, CHD, anti-cancer, antioxidant and antibacterial as keywords to review the information about botany, ethnopharmacology, phytochemistry, pharmacology, quality control and toxicology studies of AMB.

## 3. Geographical Distribution and Botany

**Figure 1:**
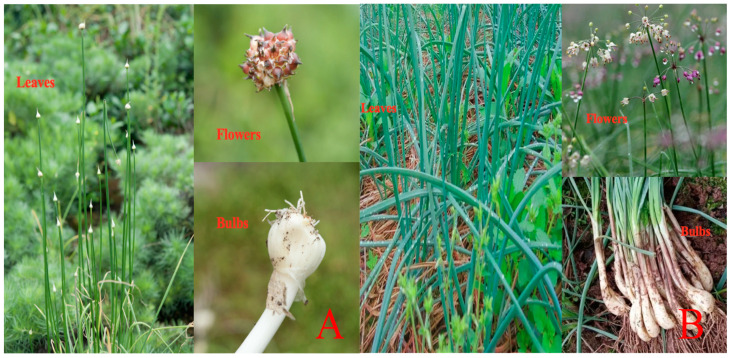
Plants form of *A. macrostemon* (**A**) and *A. chinense* (**B**) (www.gbif.org, accessed on 18 April 2022.).

Most of the *Allium* spp. of the family Liliaceae are distributed in the Northern Hemisphere, mainly in the Asia region, with about 660 species. Of these, 138 species grow in China, including 50 endemic varieties and 5 introduced varieties. Most varieties grow in arid areas, but a few species grow in ditch-side forests or watery meadows. *A. macrostemon* is distributed in all provinces and regions of China (except Xinjiang and Qinghai), mainly on mountain slopes, hills, valleys or grasslands at altitudes below 1500 m, and a few on mountain slopes at altitudes up to 3000 m (Yunnan and Tibet), and also in Russia, Korea and Japan. *A. chinense* is widely cultivated in the Yangtze River basin and provinces and regions south of China. It is also cultivated in Japan, Vietnam, Laos, Norway, and the United States [3,4]. *A. macrostemon* and *A. chinense* are seasonal wild vegetables; their leaves are typically eaten in late spring and early summer, while the bulbs hidden underground are savored in late summer and early autumn. They are very similar in appearance and morphology. Both of them usually have 2–5 hollow leaves and cylindrical scapes; the involucre is 2-lobed, with umbels. Both have depressed nectaries and are covered at the base by cap-like projections; the styles extend beyond the perianth. The differences between the two in terms of plant appearance and morphology are shown in Table 1 and Figure 1.

## 4. Ethnopharmacology

AMB was first recorded as a medicinal herb to treat weapon injuries and anti-fatigue in *Shennong Bencao Jing* compiled in the Eastern Han Dynasty; in the Tang Dynasty’s *Qianjin Yi Fang* and *Bencao Shiyi*, AMB was used to treat chest paralysis and heart pain, and to stop diarrhea and remove dysentery. With the development of the times and the advancement of science, AMB is also considered to be useful in the treatment of CHD, sudden death, nodules, stroke, burns, diarrhea, dysentery, cough and asthma, calming the fetus, and detoxification. From ancient times to the present, the concoction of AMB has also undergone a process from simple to complex (Table 2).

Since ancient times, AMB has been used in several formulas, as shown in Table 3, the most famous of which are the classical formulas mentioned in Zhang Zhongjing’s “*Jin Gui Yao Lue*” during the Eastern Han Dynasty, including Gualuo Xiebai Baijiu Decoction, Gualou Xiebai Banxia Decoction and Zhishi Xiebai Guizhi Decoction. Nowadays, these classical formulas of AMB as the “monarch drug” have been developed into proprietary Chinese patent medicines for clinical application. In addition, many proprietary Chinese patent medicines containing AMB have been developed in different dosage forms, such as Xuezhitong-capsules (XZT), Xiebai-powder, Tongxiening -granules, Dan-Lou-tablets, and Zhenxintong-oral liquid.

## 5. Phytochemistry

AMB is extremely rich in phytochemicals and has been shown to contain steroidal saponins, flavonoids, phenylpropanoids, alkaloids, volatile oils, polysaccharides, organic acids, amino acids, etc. Different methods of preparation and extraction have a great influence on the content of active ingredients in AMB, and can even change its physicochemical properties, thus affecting the therapeutic effect [2]. 

### 5.1. Steroids and Steroidal Saponins

**Figure 2:**
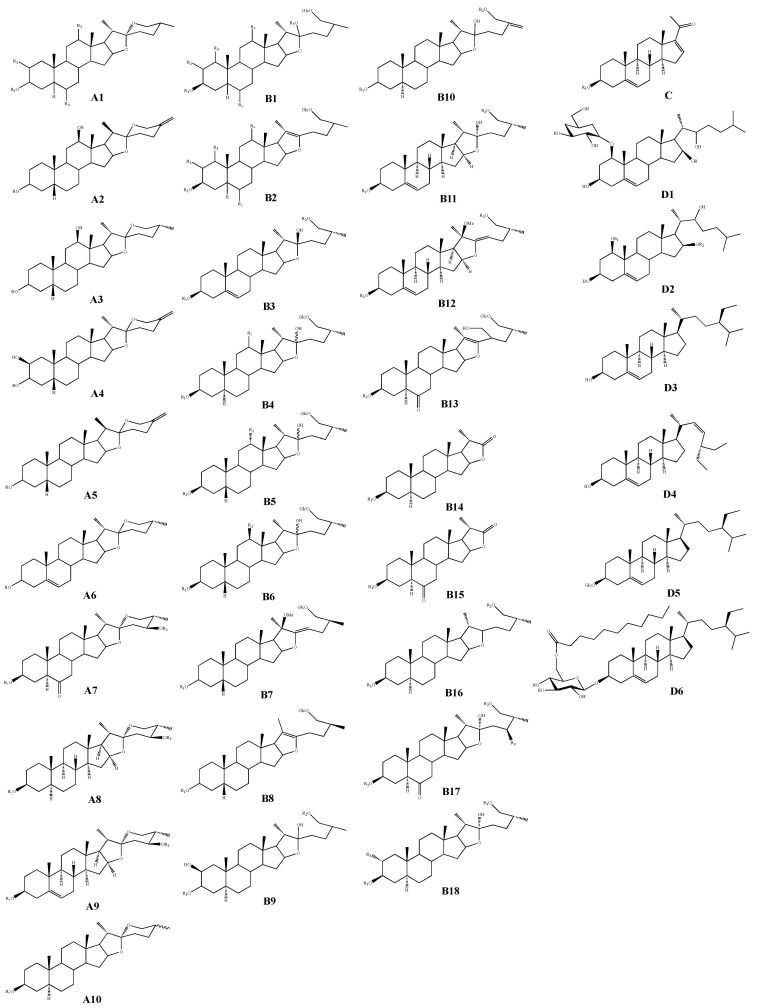
Structure of steroids and steroidal saponins isolated from AMB.

Steroids are a general term for compounds with a steroid parent nucleus, i.e., a cyclopentanoperhydrophenanthrene carbon skeleton. The physiological function of steroid compounds depends on the type and number of functional groups attached to the core ring and the configuration of the positions [5,6]. Steroid saponins are one of the main active substances in AMB; the parent nucleus is mainly of two types, spirostanol and furostanol, and the sugar part is mainly glucose, galactose, xylose, arabinose, and other monosaccharides. The sugar chain is usually attached to the C-3 position of spirostanol saponins, C-3 and C-6 positions of furostanol saponins, and to the C-1, C-6, C-12, and C-24 positions of steroid saponins; their structural diversity contributes to their wide range of pharmacological activities. Since the isolation of furostanoside and chinenoside I (**54**) [7] from *A. chinense* in 1989, a total of 89 steroidal saponins have been isolated and obtained, including spirostanosides (**1**–**28**) and furostanosides (**29**–**89**), in addition to pregnane glycoside (**90**) and cholestane glycosides (**91**–**92**), sitosterol (**93**), stigmasterol (**94**), daucosterol (**95**), sitosteryl-6’-O-undecane-β-D-glucoside (**96**), etc. The structures are shown in Table 4 and Figure 2.

### 5.2. Volatile Oils and Sulfur-Containing Components

**Figure 3:**
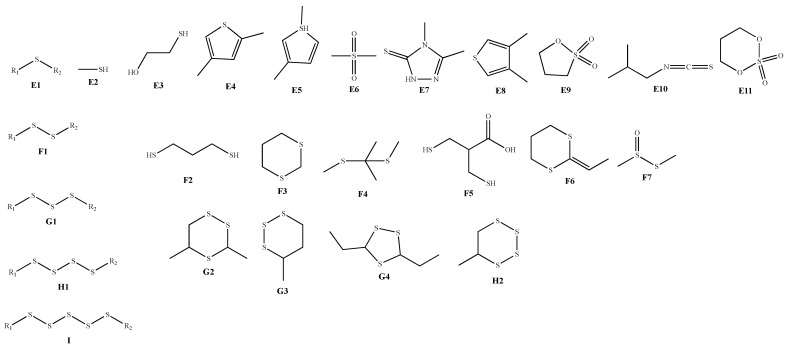
Structure of sulfur-containing compounds identified in AMB.

The special odor of AMB originates from the sulfur-containing compounds in the volatile oil, which constitute over 50% [28]. Most of the sulfur-containing compounds contain 1–5 S atoms in their molecules, characterized by the combination of different aliphatic side chains or rings on the sulfur skeleton. Some scholars used GC-MS to analyze the volatile oil of AMB and identified 14 chemical components, of which sulfur-containing compounds accounted for 93.46% [33]. Interestingly, the composition and proportion of sulfur-containing compounds identified in the volatile oil of AMB from different origins varied considerably, which may be related to the origin of AMB, but all contained methyl allyl trisulfide (**139**) [34]. In addition, there were differences in the chemical composition of volatile oils and their relative contents before and after AMB concoction. A total of 13 and 20 compounds were identified in the bulbs and leaves of fresh AMB, accounting for 62.5% and 59.63% of the total volatile oils, respectively; a total of 9 and 13 compounds were identified in the bulbs and leaves of AMB dried in an oven at 50 °C after steaming, accounting for 74.89% and 87.66% of the total, respectively [35]. The structures of the sulfur-containing compounds are shown in Table 5 and Figure 3**.**

### 5.3. Nitrogen-Containing Components

**Figure 4:**
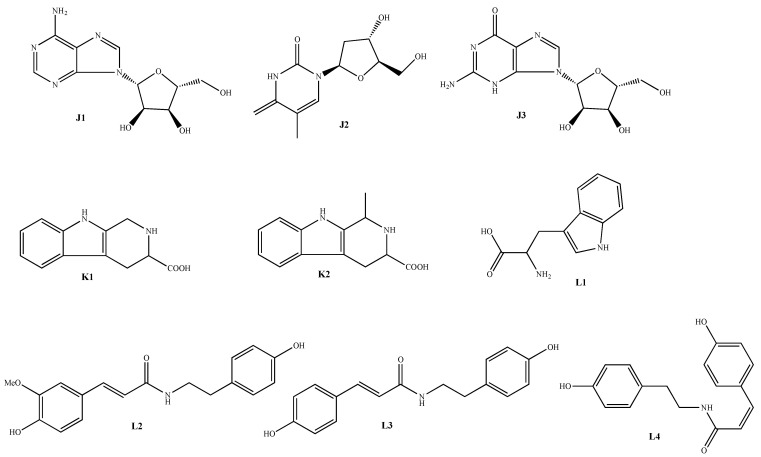
Structure of nitrogen-containing components isolated from AMB.

Nitrogen-containing compounds are also one of the main active substances in AMB. Adenosine (**155**) has been developed as an antiarrhythmic drug and was approved for use by the FDA in 1989. Adenosine (**155**) is present in large amounts in AMB and has strong platelet inhibitory activity [42]; therefore, the development of anti-arrhythmic drugs that are associated with AMB can be considered. In addition, endogenous nucleosides similar to adenosine (**155**) were identified, including thymidine (**156**) and guanosine (**157**), and other active ingredients were N-*trans*-feruloyltyramine (**161**), N-(*p*-*cis*-coumaroyl)-tyramine (**163**) and its *trans*-enantiomer (**162**), 2,3,4,9-tetrahydro-1H-pyrido [3, 4-b]indole-3-carboxylic acid (**158**) and its 1-methylated product (**159**) and tryptophan (**160**), etc. [43,44,45]. In addition, AMB is rich in many free amino acids, including 19 common protein amino acids such as arginine, threonine, serine, and 4 non-protein amino acids [46]. The structures of the nitrogen-containing compounds are shown in Table 6 and Figure 4.

### 5.4. Phenylpropanoids

**Figure 5:**
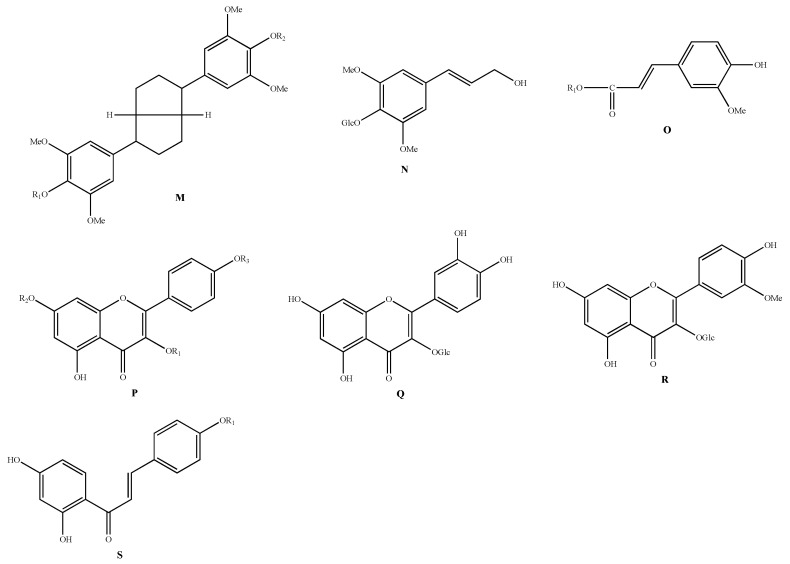
Structure of phenylpropanoids and flavonoids isolated from AMB.

Phenylpropanoids are a naturally occurring class of compounds consisting of a benzene ring linked to three straight chain carbons (C6–C3 groups). In biosynthesis, most of these compounds are formed from anthranilic acid through a series of reactions such as deamination and hydroxylation by aromatic amino acids such as phenylalanine and tyrosine. Phenylpropanoids found in AMB include acanthoside D (**164**) [48], syringin (**165**) [42], In the leaves of AMB allimacronoid A (**166**) allimacronoid B (**167**), allimacronoid C (**168**), allimacronoid D (**169**), tuberonoid A (**170**), 1-O-(E)-feruloyl-β-D gentiobioside (**171**), 1-O-(E)-feruloyl-β-D-glucopyranoside (**172**), and *trans*-ferulic acid (**173**) [49,50]. The structures of phenylpropanoid compounds are shown in Table 7 and Figure 5.

### 5.5. Flavonoids

Flavonoids are a general term for a class of compounds derived from 2-phenylchromone as a backbone. Flavonoids in AMB are mainly flavonol glycosides and chalcones, including kaempferol-3-O-β-D-glucoside (**174**), kaempferol-3,7-O-β-D-diglucoside (**175**), kaempferol-3,4’-O-β-D-diglucoside (**176**), quercetin-3-O-β-D-glucoside (**177**), isorhamnetin-3-O-β-D-glucoside (**178**), isoliquiritigenin (**179**) and isoliquiritigenin-4-O-glucoside (**180**) [14,51]. The structures of the flavonoids are shown in Table 7 and Figure 5.

### 5.6. Polysaccharides

Polysaccharides are polymers of multiple monosaccharides linked by glycosidic bonds and are classified as homopolysaccharides and heteropolysaccharides. AMB contains a large number of polysaccharides. One study conducted acid hydrolysis tests on the three refined polysaccharides PAM-Ib, PAM-IIa and PAM-III’ from AMB and showed that all three polysaccharides contained galactose and glucose [52]. Another study used enzymatic hydrolysis of AMB polysaccharides, and the results showed that the monosaccharides included arabinose, glucose, rhamnose, and galactose [53]. Both AMP40N and AMP40S are polysaccharides isolated from AMB; AMP40N consists of arabinose and glucose, while AMP40S consists of rhamnose, arabinose, glucose and galactose and a certain amount of uridine monophosphate [54]. It can be seen that there are great differences in the monosaccharide composition, glycosidic bond type, uronic acid content and properties of AMB polysaccharides obtained by different extraction methods, but most of them are polymerized with glucose, galactose, rhamnose and arabinose. Due to the complexity of polysaccharide structure and the limitation of research means, the research into polysaccharides lags far behind other types of compounds, and only some of the fungus polysaccharides are used in clinical practice. Therefore, the research on polysaccharide components in AMB should be increased, and the relationship between structure and function of AMB polysaccharides and their mechanism of action in vivo should be dissected.

### 5.7. Other Components

**Figure 6:**
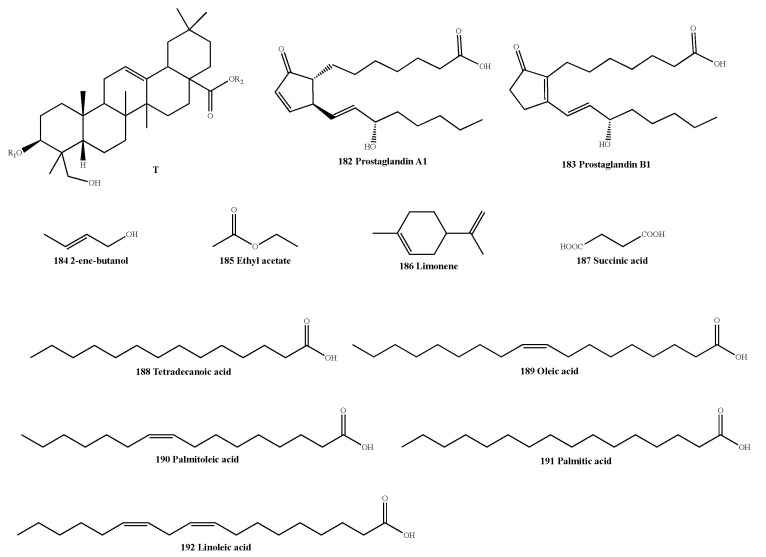
Structure of miscellaneous compounds isolated from AMB.

Other compounds isolated from AMB include (3β, 4α)-Olean-12-en-28-oic acid-3-O-β-D-galactopyranosyloxy-23-hydroxy-6-O-β-D-xylopyranosyl-β-D-galactopyranosyl ester (**181**), prostaglandin A1 (**182**), prostaglandin B1 (**183**), 2-ene-butanol (**184**), ethyl acetate (**185**), limonene (**186**) [36,41,55,56] and several fatty acid analogues, including succinic acid ( **187**), tetradecanoic acid (**188**), oleic acid (**189**), palmitoleic acid (**190**), palmitic acid (**191**) and linoleic acid (**192**) [37,40,57], whose structures are shown in Table 8 and Figure 6.

## 6. Pharmacological Activities

**Figure 7:**
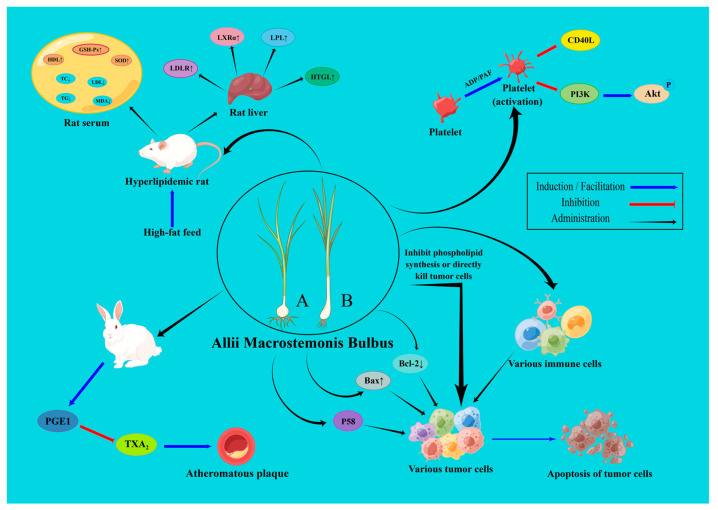
The pharmacological mechanism of AMB (Partial): *A. macrostemon* (**A**) and *A. chinense* (**B**). (www.figdraw.com, accessed on 28 February 2023).

Studies have shown that crude extracts of AMB, monomeric components (e.g., macrostemonosides), and their compound preparations exert various pharmacological activities. Some of the pharmacological mechanisms are shown in Figure 7.

### 6.1. Anti-Platelet Aggregation Effect

Adhesion, aggregation and secretion are the three basic functions of platelets. Excessive platelet activation caused by pathological factors can promote platelet aggregation, which can cause thrombotic disease [58]. In recent years, much attention has been paid to the role of platelet-associated inflammation in the pathogenesis of coronary artery disease. The release of CD40L after platelet activation and adhesion between platelets and neutrophils is one of the initiating links of thrombosis [59]. Recent studies have suggested that platelets are involved in hemostasis and thrombosis, but also secrete various inflammatory factors such as adhesion molecules (Intercellular adhesion molecule-2), P-selectin and its ligand (P-selectin glycoprotein ligand-1), which have a direct chemotactic effect on leukocytes in blood vessels and regulate the development of inflammation [60]. Inflammation contributes to vulnerable plaque thrombosis and plays an important role in the pathogenesis of acute coronary syndrome (ACS). It was found that steroidal saponins in AMB inhibit platelet CD40L expression and platelet neutrophil adhesion [23]. AMB saponins inhibit arachidonic acid (AA), adenosine diphosphate (ADP) and platelet activation factor (PAF) induced platelet aggregation in a concentration-dependent manner in vitro and in vivo, reduce intra-platelet calcium ion concentration and adhesion between neutrophils and thrombin-activated platelets, and inhibit platelet aggregation induced by neutrophil supernatant [61]. N-*trans*-feruloyltyramine (**158**), isolated from AMB, showed significant inhibition of both the first and second phases of ADP-induced human platelet aggregation, whereas N-(*p-cis*-coumaroyl)-tyramine (**160**) inhibited only the first phase of aggregation [62]. Furosterosides in AMB reduce cardiomyocyte injury in SD rats both in vitro and in vivo by inhibiting platelet phosphatidylinositol 3-kinase/proteinserine-threonine kinase (PI3K/Akt) signaling pathway and thereby inhibiting ADP-induced platelet aggregation [63]. Methyl allyl trisulfide (**136**), a sulfur-containing compound in AMB, showed strong inhibition of platelet aggregation activity [37,41]. Given the relationship between platelets and inflammatory factors, it is suggested that the relationship between the pharmacological effects of AMB and inflammation is also one of the directions worth investigating.

### 6.2. Hypolipidemic and Anti-Atherosclerotic Effects

Atherosclerosis (AS) is a chronic inflammatory disease caused by impaired lipid metabolism, usually forming plaques in medium and large arteries [64], and is a major cause of the development of CHD and cerebral vascular accident (CVA) [65]. The accumulation of macrophages under the endothelium is thought to be the first step in the formation of AS, and over time, atherosclerotic plaques become more fibrotic and cause calcium deposits, which can eventually invade the lumen and lead to the development of ischemic disease [66].

Mammalian target of rapamycin (m TOR) is a serine/threonine protein kinase found in mammals and has important roles in cell proliferation, survival, metabolism, autophagy, apoptosis, migration, and other biological processes. Several studies have shown that m TOR activation triggers endothelial dysfunction, foam cell formation, and vascular smooth muscle cell proliferation, thereby promoting the development and progression of AS [67,68]. Furthermore, in the early stages of atherosclerosis, low-density lipoprotein (LDL) is retained in the intima and is modified to form multiple danger-associated molecular patterns (DAMP), mediated by oxidases, lipolytic enzymes, protein hydrolases, and reactive oxygen species, thereby acquiring immunogenicity [69], and immunogenic LDL activates vascular endothelial cells. Vascular endothelial cells regulate the structure and function of blood vessels by releasing biochemical factors such as nitric oxide (NO) and prostaglandin I2 (PGI2) [70].

It was found that AMB total saponin and volatile oil extract could significantly reduce serum and liver total cholesterol (TC), triglyceride (TG), and LDL levels, and increase serum high-density lipoprotein (HDL) levels in rats on a high-fat diet, thus exerting a hypolipidemic effect [71,72]. One of the possible mechanisms for AMB to lower lipids and prevent atherosclerosis is to increase the levels of PGI2 and PGE1 on the one hand and to interfere with AA metabolism and inhibit thromboxane A_2_ (TXA_2_) synthesis, on the other hand, thus changing the PGI2/TXA2 ratio and relieving the hypercoagulable state of blood [73,74]. Another study showed that 10% AMB powder added to the high-fat diet of an animal with hyperlipidemia could upregulate the mRNA expression levels of low-density lipoprotein receptor (LDLR) and liver X receptor alpha (LXRα) in liver tissue, thus exerting its hypolipidemic effect [75]. Macrostemonoside A (**1**) is a steroidal saponin isolated from AMB, which can reduce TC, TG, and LDL levels in mice serum and blood glucose levels in mice, and increase visfatin protein expression in 3T3-L1 cells [76,77]. XZT is a proprietary Chinese patent medicine made from AMB extract. Studies have shown that XZT reduces fatty acid synthase (FAS) and LDL levels in the serum of ApoE^−/−^ mice by activating reverse cholesterol transport (RCT) and increasing HDL levels, and that XZT reduces TG levels in patients with hyperlipidemia [78,79]. The m TOR signaling pathway plays an important role in the progression and treatment of CHD. m TOR is mostly associated with cellular autophagy and apoptosis, and previous studies have demonstrated that autophagy has a dual role in atherosclerosis. The body needs moderate autophagy to stabilize plaque and inhibit excessive autophagy during cardiac I/R injury to reduce myocardial infarct size. Most of the monomeric components of TCM for the treatment of CHD are purified from blood-stasis-activating and qi-supplementing drugs, but the mechanisms of pharmacological effects of qi-activating drugs (e.g., AMB) and expectorants (e.g., Fructus Trichosanthis and Pinellia Tuber), which are commonly used in the clinical treatment of CHD, have been less studied, and research on the mechanisms of active components of these herbs should be strengthened.

### 6.3. Protection of Cardiomyocytes and Vascular Endothelial Cells

Myocardial ischemia is the result of an imbalance in oxygen supply and demand to myocardial cells, and early hemodialysis is the most effective way to reduce post-ischemic myocardial injury [80]. With the development of the application of interventions such as percutaneous coronary intervention, coronary artery bypass grafting, and thrombolysis, the myocardium can be resupplied with blood after ischemia, but the ensuing myocardial ischemia-reperfusion injury is a complex pathophysiological process involving multiple factors. The mechanism is currently believed to be closely related to inflammation, oxidative stress, vascular endothelial cell damage, platelet aggregation, and other factors, which can eventually lead to irreversible apoptosis or necrosis [81,82,83]. Early reperfusion therapy can aggravate the myocardial injury and become an important factor affecting the outcome of ischemic therapy. The assessment and treatment of reperfusion injury remain a clinical challenge, and the causal mechanism is still unclear. One mechanism that has been identified is that ischemia-reperfusion triggers endothelial cell dysfunction and disrupts the endothelial structure of the blood vessels, thereby impeding blood circulation within the microvasculature. Endothelial cells are not only found in the lining of blood vessels but also cover the heart and lymphatic lumen longitudinally in a single layer, playing an important role in normal cardiac physiology and cardiac response to injury. Endothelial cells also act as secretory cells, secreting vasoactive substances, such as the vasoconstrictors endothelin (ET) and angiotensin, and vasodilators such as NO and endothelial-dependent hyperpolarizing factor (EDHF). They play an important role in regulating the tone of blood vessels, especially microcirculatory vessels; they can also synthesize and secrete relevant coagulation factors and fibrinolytic substances to maintain a dynamic balance between coagulation and fibrinolysis and influence the coagulation and fibrinolysis process, thus maintaining normal blood flow and circulation [84]. It was found that AMB extract reduced the gene expression of inflammation-related cyclooxygenase-2 (COX-2), cyclooxygenase-1 (COX-1), inducible nitric oxide synthase (iNOS), and vasodilation-related endothelin-converting enzyme (ECE), and endothelial nitric oxide synthase (eNOS), but increased the gene expression of antioxidant superoxide dismutase (SOD) in a model of air-stressed vascular endothelial injury, thereby reducing endothelial vascular damage in model rats [85,86]. At the same time, AMB extract also significantly reduced plasma ET level, increased serum NO level, and inhibited glucose-regulated protein 78 (GRP78) protein expression in aortic tissue to improve vascular endothelial function in model rats by suppressing endoplasmic reticulum stress [87]. In a rat model of acute myocardial ischemia caused by open-chest ligation of the anterior descending branch of the rats’ left coronary artery, ethanolic extract of AMB can regulate the balance of lipid and protein metabolism and reduce the damage caused by acute myocardial ischemia in the rat organism [88]. AMB extract also significantly increased serum glutathione peroxidase (GSH-Px) activity; it decreased acetylcholinesterase (TChE) activity, non-esterified fatty acid (NEFA), and malondialdehyde (MDA) content, and reduced the extent of myocardial injury in rats [89]. In addition, AMB extracts could protect vascular endothelial function in depressed rats by enhancing 5-hydroxytryptamine 1D (5-HT_1D_) mRNA and protein expression, which mediates the diastolic effect, and inhibiting 5-hydroxytryptamine 2A (5-HT_2A_) mRNA and protein expression, which mediates the vasoconstrictive effect [90].

### 6.4. Anti-Cancer Effect

In medicine, cancer is defined as a malignant tumor often originating from epithelial tissue, which is the most common type of malignancy. Globally, cancer has become the leading cause of human death and a serious obstacle to increasing human life expectancy [91]. Today, global cancer incidence and mortality rates are increasing every year, with 28.4 million cancer cases expected in 2040 [92]. The anti-cancer activity of AMB is mainly related to the water-soluble saponins, polysaccharides, and fat-soluble volatile oils contained in it. Reports have illustrated that the active components in AMB have been effective against human non-small cell lung cancer A549 [13,30], human lung cancer cells PC-9 [13], mice sarcoma cells S180 [93,94], mice liver cancer cells H22 [94], human gastric cancer cell SGC-7901 [95], human breast cancer MCF-7 [21], human neural cancer cell SF-268 [21,25,96], human lung cancer cells NCI-H460 [21,25,96], human cervical cancer HeLa cells [14,97,98], human colon cancer cells SW-480 [99], mice melanoma cells B16 [100], mice breast cancer cells 4T1 [100], human hepatoma cells Hep-3B [101], human hepatoma cells HepG2 [21,30,97], human lung adenocarcinoma cell SPC-A-1 [30], human gastric cancer cell MGC80-3 [30], human breast cancer cell MDA-MB-231 [30], human colon cancer cell SW620 [30] and human nasopharyngeal carcinoma cells CNE-1 [30], which were inhibited in vivo or in vitro. Possible mechanisms of action include: regulation of EGFR/PI3K/m TOR and RAF/MAPK signaling pathways [13]; inhibition of tumor cell membrane phospholipid synthesis [14]; enhancement of immune function in mice, especially cellular immune function, which is dominant in tumor immunity, and thus suppression of tumor cells [93,100]; directly killing tumor cells by destroying nuclei and organelles [94]; altering the G_2_/M cell cycle of tumor cells [30,97]; promoting the expression of P53 protein to induce apoptosis [94,95]; decreasing mitochondrial membrane potential; up-regulating Bax mRNA expression, down-regulating Bcl-2 mRNA expression, and Bcl-2/Bax ratio; enhanceing Caspase-9 and Caspase-3 activity; inducing reactive oxygen species (ROS) production, and promoting apoptosis of tumor cells [98,99,101].

### 6.5. Antibacterial Effect

The extracts of AMB have inhibitory effects on a variety of bacteria and fungi. It was found that the aqueous extract of AMB had a wide range of antibacterial abilities, and the antibacterial ability varied at different dilutions of the extracts, with a more desirable effect at higher concentrations, and weaker effect at higher dilutions [102]. In addition, the ethanol extract of AMB also has an inhibitory effect on most bacteria, and the inhibition ability is influenced by temperature and pH. The strongest inhibition activity is at 50–60 °C and the activity decreases when the temperature is greater than 100 °C. The inhibition activity is stronger when the pH is neutral or nearly neutral, and the inhibition activity gradually decreases with the enhancement of acidity or alkalinity [103]. AMB may exert its bacterial inhibitory effect by inhibiting the synthesis of bacterial-associated proteins, inhibiting the activity of related enzymes, or changing their cell structure [104,105]. The material basis of these mechanisms may be related to the sulfur-containing compounds in AMB, and the specific mechanism of action needs to be investigated in depth.

### 6.6. Anti-Asthmatic Effect

Asthma, as a chronic inflammatory disease of the respiratory tract, is one of the most common non-communicable diseases of the respiratory system in children and adults, often caused by allergic reactions. Stimuli such as histamine, acetylcholine, or cold air can cause airway hyperreactivity and produce airway obstruction, which can clinically cause recurrent episodes of wheezing, chest tightness, or coughing [106]. Typical asthma pathology is characterized by airway inflammation, smooth muscle contraction, epithelial cell shedding, excessive mucus secretion, bronchial hyperresponsiveness, and mucosal edema [107]. Standard therapies for asthma are mainly based on bronchodilators and immunosuppressive drugs, which provide short-term relief but not a cure. Chinese medicine has played an important role in the treatment of various respiratory diseases, including asthma, and has a history of more than 2000 years in the treatment of asthma. In recent years, more and more researchers have focused on the effects of Chinese medicine on asthma, and have achieved remarkable results in clinical trials or basic experimental models [108,109]. Clinically, AMB can be used alone for the treatment of asthma, and in recent years, many studies have been conducted on the pharmacodynamic material basis of AMB for the treatment of asthma. It has been reported that in animal experiments, IL-6 mRNA content in the bronchial tissues of asthmatic guinea pigs was significantly increased [110,111]. In clinical practice, serum levels of IL-6 are also significantly higher in asthmatics than in normal subjects [112,113,114]. In addition, the balance of TXA_2_ and PGI2 is an important regulatory mechanism in the pathophysiological mechanism of asthma, and if the ratio of TXA_2_/PGI2 is increased, it causes bronchial smooth muscle contraction leading to asthma; however, because of the instability of TXA2 and PGI2, the corresponding metabolites of both, thromboxane B_2_ (TXB_2_) and 6-keto-prostaglandin F_1α_ (6-Keto-PGF_1α_) are often measured [115,116,117]. Studies have shown that AMB extract can reduce the expression levels of IL-6 and TXB_2_ and up-regulate the expression level of 6-Keto-PGF_1α_ in the serum of asthmatic guinea pigs, thus achieving a panting effect [118]. In vivo and in vitro, the active ingredients in AMB effectively diastole bronchial smooth muscle in a guinea pig model of histamine-induced asthma [119,120]. In summary, we deduce that the mechanism by which AMB exerts its effect on wheezing may be through the inhibition of inflammatory response, alleviating chronic inflammation and thus relieving the spastic state of bronchial smooth muscle.

### 6.7. Antioxidant Effect

ROS are oxygen-containing radicals with high oxidative capacity and high activity generated during metabolism, mainly including superoxide anion radical (O_2_^−^), hydrogen peroxide (H_2_O_2_), hydroxyl radical (·OH), etc. ROS are a double-edged sword for cellular life activities: on the one hand, ROS are important tools or signaling molecules in specific cells (such as macrophages, etc.) and play an important role in removing pathogenic microorganisms, maintaining the normal vascular function, and regulating intracellular homeostasis. On the other hand, when the excessive production of intracellular ROS exceeds the scavenging capacity of the antioxidant system in the body, they will attack proteins, DNA and lipids, causing oxidative stress, which is one of the important factors in the occurrence of cell damage, inflammation, and metabolic disorders [121,122,123,124]. Antioxidant enzymes in the body mainly include SOD, GSH-Px, glutathione S-transferase (GST), catalase (CAT), etc. Non-enzyme antioxidants include glutathione, vitamin E, vitamin C, etc. SOD can effectively scavenge O_2_^−^, protect cells from oxidative damage, and also provide hydrogen atom ligands for the reduction of ROS to produce hydrogen peroxide, which in turn can be catalyzed by GSH-Px and CAT to produce water and oxygen to reduce oxidative stress damage [125,126,127]. Oxidative stress is associated with multiple signaling pathway molecules. Nuclear factor erythroid 2-related factor 2 (Nrf2) is a basic leucine zipper transcription factor, and cytoplasmic Nrf2 is normally bound to Kelch-like ECH-associated protein-1. The free Nrf2 is able to translocate from the cytoplasm to the nucleus, where it forms a heterodimer with Maf family proteins and then binds to antioxidant response element sequences to induce the expression of downstream antioxidant enzymes, thereby scavenging ROS, inhibiting oxidative stress, maintaining the structural integrity and normal metabolic function of the cell, and exerting its transcriptional regulatory role [128,129,130,131]. Nuclear factor kappa-B (NF-κB) is a dimeric protein of the Rel family. The heterodimer composed of p65 and p50 is a common activated form of NF-κB. NF-κB can promote the infiltration of neutrophils and macrophages and the release of cytokines, chemokines, adhesion molecules, etc., stimulate the expression and secretion of matrix metalloproteinases, activate nicotinamide adenine dinucleotide phosphate oxidase to produce large amounts of ROS, and trigger oxidative stress-related inflammatory diseases [132,133,134]. Silent information regulator 1 (Sirt1) is a nicotinamide adenine dinucleotide-dependent deacetylase. Activated Sirt1 inhibits p66shc expression and reduces mitochondrial ROS production by regulating p66shc, which deacetylates histone H3 bound to the p66shc promoter [135,136,137]. It is found that AMB extract alleviates liquor-induced oxidative stress in rats by increasing serum SOD and CAT activities and protecting T lymphocytes, and significantly inhibiting serum lipid peroxide formation [138]. AMB polysaccharide, AMB saponin, and some sulfur-containing compounds can effectively scavenge DPPH, O_2_^−^ and ·OH in vitro and inhibit the oxidation of Fe^2+^ to a certain extent, and their antioxidant ability can be enhanced after modification with chlorosulfate-pyridine or α-amylase for AMB polysaccharide [139,140,141,142,143]. Although there are many experimental studies on the antioxidant activity of various extracts of AMB, most of them are limited to in vitro experiments and the specific mechanism is not yet clear. The research efforts on oxidative stress signaling molecules should be deepened to elucidate the antioxidant mechanism of AMB at the molecular level.

### 6.8. Antidepressant Effect

Depression is an affective disorder characterized by persistent mood abnormalities, mainly manifested as depressed mood, lack of pleasure, difficulty concentrating, fatigue, physical pain, and other symptoms, with a high disability rate and high patient suicide rate, which brings a serious economic burden to the patient’s family and society [144,145]. The pathogenesis of depression has not yet been fully investigated and researchers have proposed various hypotheses, among which the monoamine transmitter theory suggests that the development of depression is mainly due to the reduction of 5-hydroxytryptamine (5-HT) and norepinephrine (NE) in the brain; therefore, inhibiting the degradation and reuptake of these two monoamines is beneficial to improve depressive symptoms [144]. The neurotrophic factor hypothesis focuses on the brain-derived neurotrophic factor (BDNF) and suggests that an imbalance of brain derived neurotrophic factor precursor (proBDNF) and mature form of brain-derived neurotrophic factor (mBDNF) is closely related to the development of depression [146]. The neurogenesis hypothesis suggests that downregulation of hippocampal neurogenesis is the cause of depression and that antidepressants work based on promoting neurogenesis [147,148]. In addition, possible mechanisms such as the hypothalamic-pituitary-adrenal (HPA) axis dysregulation hypothesis, inflammation hypothesis, and genetic hypothesis have also been proposed to explain the development of depression [149]. Depression is gradually becoming an important health problem faced by all human beings today, and its pathogenesis is complex. Although antidepressant western drugs are effective for patients with critical symptoms, they have more side effects in terms of mental and emotional effects when taken for a long time. Therefore, people gradually turn their horizons to Chinese medicine, but the composition of Chinese medicines is complex. It can be difficult to find the best component with significant efficacy among the complex and numerous components of compound medicines and single component treatments. The mechanism by which AMB exerts antidepressant effects on various animal models of depression (including rats and mice) may be through regulating the balance of the internal environment of depression model animals, promoting neurogenesis and BDNF production; at the same time, AMB can significantly improve the pathological changes of organ tissues in the relevant animal models [150,151]. In addition, the analysis of lipids and acylcarnitine in the plasma of depressed rats by liquid chromatography/ion trap time of flight mass spectrometry and ultra-performance liquid chromatography/triple quadrupole mass spectrometry, respectively, showed that the AMB aqueous extract was able to restore the normal levels of these abnormally altered indicators [152]. Although there are numerous studies on depression, the relevant mechanisms are still under-explained, and more rigorous experimental design is needed in the future, together with modern technology to reduce complex Chinese medicine into simpler groupings, purify components, or increase the study of mechanisms at the cellular-molecular level. It cannot be ignored, however, that Chinese medicine mostly follows a certain idea of combination, and it is necessary to maintain a cautious attitude whether the antidepressant components derived from the reductionist ideas of modern medicine can stand up to clinical tests.

### 6.9. Other Pharmacological Effects

In addition to the above pharmacological effects, AMB and its compounds exhibit other activities such as analgesia, hypoxia tolerance, immunomodulation, promotion of osteogenesis, inhibition of hepatic drug enzymes, and mosquito control. Studies have shown that both the raw aqueous decoction of AMB and its fried aqueous decoction have strong analgesic effects and prolong the duration of hypoxia tolerance in mice with enhanced oxygen consumption induced by NaNO_2_ intoxication and isoproterenol (ISO) under normoxic conditions. The mechanism of analgesia of AMB may be through the inhibition of voltage-sensitive Nav1.7 channels, thus reducing the excitability of peripheral neurons and exerting analgesic effects [153,154]. AMB can increase the weight of mice’s immune organs, the spleen and thymus, and can increase carbon particle contouring index K and phagocytosis index α; that is, it can promote the phagocytosis of mononuclear macrophages and improve the specific immune function of the body. AMB volatile oil can increase the spleen index, macrophage phagocytosis rate and splenocyte proliferation index. The regulatory ability of AMB on the immune system may be one of the mechanisms of its anti-tumor effect [93,155]. AMB alcohol extract can increase the expression of insulin-like growth factor-1 and bone morphogenetic protein-2, thus regulating the formation and resorption of bones and achieving the purpose of promoting bone growth [156]. AMB aqueous extract can significantly reduce the content of cytochrome P450 in mice and has a significant inhibitory effect on hepatic drug enzymes [157]. In addition, the volatile oil of AMB and its two main components (compounds **113** and **135**) exhibited strong larvicidal effects against *Aedes albopictus* larvae, suggesting the existence of a basis for the development of mosquito control agents [158]. The modern pharmacological studies on AMB are summarized in Table 9.

## 7. Quality Control

The quality control of Chinese medicine is a prerequisite to ensuring the safe and effective clinical application of Chinese medicine. Standardized research on the quality of Chinese medicine is the top priority to achieve the sustainable development of Chinese medicine in recent years, and strengthening the quality control of Chinese medicine is of great significance to ensure the safety of people’s medicine and promote the development of the Chinese medicine industry. In the 2020 Edition of the ChP, the quality control of AMB mainly includes microscopic identification, thin-layer chromatography (TLC), moisture, total ash, and ethanol leachate detection, and states that the moisture content of AMB shall not exceed 10.0% by the toluene method, the total ash content should not exceed 5.0% by constant weight method, and the leachate content obtained by heating extraction with 75% ethanol shall not be less than 30.0% [1]. It was reported that the surface-enhanced Raman scattering (SERS) spectra of AMB volatiles of different species from different production areas were tested with nano-silver sol as the substrate. The results showed that the SERS spectra of these batches of AMB volatiles were very similar; the intensity of the characteristic peaks varied somewhat, but the peak positions were basically unchanged, and the reproducibility was good, indicating that nano-silver sol could be used as the substrate of SERS for the determination of AMB volatiles [34]. Other scholars have used chromatographic methods to study the content of each component in AMB. This includes the quantitative analysis of furostanol saponins in AMB using high performance liquid chromatography [171] and determination of adenosine (**155**) in AMB by reversed-phase high performance liquid chromatography [172]. Gas chromatography-mass spectrometry was used to qualitatively and quantitatively analyze the volatile oil of AMB, and the main components were identified as sulfur-containing compounds and their mass fractions [38]. High performance liquid chromatography-mass spectrometry was used to determine the concentration of AMB saponins in rat plasma and tissues; the experimental results showed that AMB saponins were high in rat liver and kidney, and no such components were detected in brain and lung tissues. This method provides theoretical guidance for AMB quality control and drug use, but there are shortcomings, since the experimental detection of AMB saponins monomers only selected the highest plasma exposure monomers, leaving a future need to study other monomers with high relative exposure [173]. Another study used chemometric methods to select the main components and major absorbed components in rats as their representative components. It then established ultra performance liquid chromatography coupled with quadrupole time-of-flight tandem mass spectrometry for the simultaneous determination of 54 components (15 components were quantitative and 39 components were semi-quantitative), which facilitated the screening of AMB quality markers [174]. In addition, the determination of furostanol saponins content in AMB by colorimetric method with Ehrlich reagent can also be used as one of the methods to evaluate the quality standard of AMB [175]. The main active ingredients in AMB are steroidal saponins, sulfur-containing, and nitrogen-containing compounds. So far, the quality markers of AMB are still unclear, and the current ChP does not have its quantitative standards, so deepening the screening of AMB quality markers is one of the efforts to optimize the quality control of AMB. Further research and development by scholars in this industry are needed to ensure its quality assurance and medication safety.

## 8. Toxicology

The ancient Chinese medical classics, “*Mingyi Bielu*”, states that AMB is “bitter in taste and non-toxic”, and the same is true of “*Bencao Gangmu*”, which also states that it is non-toxic. In the 2020 Edition of the ChP, the recommended daily dose of AMB for adults is 5–10 g. To date, there have been very few reports of toxicity or side effects of AMB. After reviewing the relevant literature, only one case of intestinal rumbling and diarrhea with yellow watery stools after taking AMB was found, but the specific mechanism is unclear. It is speculated that the components contained in AMB may act as antigens or semi-antigens when they enter the body to cause metabolic diseases in the body, or the components in AMB may directly stimulate mast cells or basophils to release allergic mediators (such as histamine, 5-HT, etc.) or there could be direct activation of the complement system, direct or indirect action on target organs or organs in shock [176]. The oral median lethal dose (LD_50_) of AMB and its compounds were greater be more than 100 times of their respective clinical doses, and the toxicity was very low. The LD_50_ of AMB (70.12 ± 3.49 g/kg) and the compounds (48.72 ± 1.79 g/kg) were administered intraperitoneally to mice, and the symptoms of toxicity were similar, including reduced activity, weakness of limbs, flaccidity, and convulsions [177]. In addition, AMB should be used with caution in patients with Yin deficiency and fever and Qi deficiency, and it is said that AMB should not be consumed with beef. In summary, AMB can be considered non-toxic, with the possibility of toxic reactions only in rare cases or in very large doses for long-term use.

## 9. Conclusions and Outlook

AMB has a long history of use. As a special herbal medicine for the treatment of “obstruction of Qi in the chest”, AMB has the efficacy of activating Yang and removing stasis, regulating Qi and eliminating stagnation, and is abundant, inexpensive, and of high medicinal value. This review systematically summarizes botany, ethnopharmacology, phytochemistry, pharmacological effects, quality control, and toxicology of AMB. Botanically, AMB has two sources, *A. macrostemon* and *A. chinense*, which are very similar and can be distinguished in the intact plant by the shape and color of the bulb, the length of the scape and pedicel. However, the dried product is difficult to distinguish from its appearance and can be distinguished by microscopic identification. The origin of the two is also different (see Figure 1). In traditional applications, AMB is often used in combination with Fructus Trichosanthis, Pinellia Tuber, Cassia Twig, etc., and is clinically effective in the treatment of CHD, AP, and other diseases. However, the efficacy of AMB in ancient Chinese medical books is not limited to this, but also includes anti-fatigue, promotion of wound healing, treatment of CVA, etc. The research into how AMB achieves such effects should be broadened, to expand its medicinal scope and give greater play to its medicinal value. In addition to its medicinal use, AMB is also included as food in the Health Law of the People’s Republic of China, and this medicinal food homologation also provides a favorable condition for further development of AMB in the future.

In recent years, the research results on AMB in phytochemistry and pharmacological effects have been remarkable. In phytochemistry, so far, more than 190 kinds of compounds have been extracted and isolated from AMB, with as many as 96 steroidal components, including 89 steroidal saponins, and also some sulfur-containing compounds, nitrogen-containing compounds, phenylpropanoids, and flavonoids. Modern pharmacological studies have shown that AMB has pharmacological activities in areas such as anti-platelet aggregation, hypolipidemia, anti-atherosclerosis, protection of cardiomyocytes and vascular endothelial cells, anticancer, antibacterial, anti-asthma, antioxidants, and antidepressant effects. According to the previous review, its importance may be summarized as follows. AMB may be used in the treatment of atherosclerosis, thrombosis, and hypertension caused by vascular endothelial cell injury and apoptosis. AMB exhibits protective effects on vascular endothelial cells along with antithrombotic and antihypertensive effects; endothelial cell injury is closely related to inflammatory response invasion and antioxidant effects, so the mechanism of endothelial cell protection by AMB may also be closely related to its anti-inflammatory and antioxidant effects. In addition, AMB can inhibit the invasion and migration of tumor cells to varying degrees, thus exerting its anti-tumor effects, and the mechanism is also related to the inhibition of platelet aggregation by AMB.

Behind the above research results, there are still deficiencies in the research on AMB: (1) Despite the large number of compounds isolated from AMB, the 2020 Edition of the ChP still only has microscopic identification and TLC, and no quality markers for AMB have been identified; therefore, there is a need to strengthen the screening of quality markers for AMB in combination with relevant studies on chemical composition and pharmacological activity, so as to ensure herb quality and drug safety. (2) Due to the still large technical difficulties in the isolation and purification of a large number of monomeric compounds, most of the current pharmacodynamic studies on AMB saponins have used the total extracts of AMB saponins, while lacking in-depth molecular mechanism studies. Therefore, obtaining sufficient monomeric compounds of AMB saponins and their modification products by chemical synthesis can provide in-depth studies on the pharmacodynamic effects and molecular mechanisms of the monomeric components and provide a theoretical basis for clinical exploration of potential precursor drugs. (3) Using histological and other techniques, and linking the material reflecting the diversity of chemical components with transcriptomics, proteomics or metabolomics reflecting the pharmacological mechanisms, can further elucidate the modern pharmacological mechanism of TCM by modern scientific means under the premise of multi-component drug incorporation and provide new scientific ideas for the modernization of Chinese medicine. Therefore, it is urgent to further investigate and confirm the various activities of AMB using new pharmacological models, and to clarify the corresponding active sites and active components. (4) The toxicological studies of AMB are relatively few, and such studies should be deepened. The corresponding toxicological studies should be conducted under the guidance of TCM theory.

In general, despite the many research findings on AMB, there are still many gaps. The top priority is the study of pharmacological activity of the monomeric components of AMB and the screening of quality markers. The information provided in this paper can help set targets for future research directions and commercial development of AMB.

## Figures and Tables

**Table 1:** Main differences between *A. macrostemon* and *A. chinense* in terms of plant morphology.

Distinctions	*A. macrostemon*	*A. chinense*
Bulbs	Subglobose with yellowish papery or membranous exine	Narrowly ovate with white or reddish membranous exine
Leaves	Semiterete and grooved, slightly shorter than the scapes	Terete and about as long as the scapes
Flowers	Hemispheric to globose, with numerous and crowded flowers, dark purple bulblets and pink or rose-red oblong-ovate to oblong-lanceolate tepals	Subhemispheric, with looser flowers and lavender to bluish-purple broadly elliptic to suborbicular tepals
Ovaries	Subglobose	Obovoid
Flowering and fruiting period	May–July	October–November

**Table 2:** Different processing methods of AMB in different periods.

Dynasty	Processing Method	Monograph
Tang Dynasty	Cut into one-inch lengths	Waitai Miyao
Song Dynasty	Wash the soil from the surface	Taiping Shenghui Fang
	Remove the green part, leaving the white part	Bencao Tujing
	Stir-fried AMB with the fat of lamb kidney	Zhenglei Bencao
	Fry AMB in vinegar to turn it charred black	Shengji Zonglu
	Remove the fibrous roots and stems, steam and dry in the sun	Zengding Weiyao Tiaobian
Ming Dynasty	Remove the green part and finely cut	Qixiao Liangfang
Modern	1. Cleaning: Pick impurities and sieve out fibrous roots and debris. 2. Cutting: After cleaning, cut into several sections and dry in the sun. 3. Stir-frying: Put clean AMB into a wok and fry over slow fire until the outer surface shows charred spots, remove and cool.	Chinese medicine sea
	Stir-fry with baijiu: For every 500 g of AMB, use 50 mL of baijiu, mix the two together, moisten slightly, and then fry in a wok over a slow fire until yellow in color.	Practical Chinese medicine processing
	Remove impurities, wash the soil, place it in a suitable container for light steaming, take it out, and dry it in the sun.	General guide to modern Chinese herbal medicine commodities
	Wash, remove the bearded root, steam through or put in boiling water and scald through, dry in the sun.	ChP (2020)

**Table 3:** Traditional uses of AMB in China.

Dynasty	Preparation/Single Medicine	Main Compositions	Traditional Uses	Monograph
Han Dynasty	AMB	AMB	Weapon injury-induced suppuration, anti-fatigue	Shennong Bencao Jing
	Gualou-Xiebai-Baijiu-Decoction	AMB, *Trichosanthes kirilowii*, Baijiu	Chest paralysis and heart pain, wheezing and cough, phlegm	Jingui Yaolue
	Gualou-Xiebai-Banxia-Decoction	AMB, *Trichosanthes kirilowii*, *Pinellia ternate*, Baijiu		Jingui Yaolue
	Zhishi-Xiebai-Guizhi-Decoction	AMB, *Trichosanthes kirilowii*, *Citrus aurantium*, *Houpoea officinalis*, Cassia twig		Jingui Yaolue
	Gualou-Xiebai-Tea	AMB, *Trichosanthes kirilowii*, Flower tea		Jingui Yaolue
Jin Dynasty	AMB	AMB	Sudden death	Mingyi Bielu
	Baizhimo-Ointment	AMB, *Angelica dahurica*, *Glycyrrhiza uralensis*,	The carbuncle has been festered	Liu Juanzi Guiyi Fang
		*Aconitum carmichaeli*, Green bamboo bark		
Tang Dynasty	Xiaobiejia-Decoction	AMB, *Trionyx sinensis*, *Scutellaria baicalensis*, *Cimicifuga foetida*, Ephedra, Antelope horn, *Cinnamomum cassia*, Almond, *Peucedanum praeruptorum*, Smoked plum	Physical weakness with edema	Beiji Qianjin Yao Fang
	Cangmi-Decoction	AMB, Rice, Mutton fat, Fragrant fermented soy beans	Cold dysentery	Beiji Qianjin Yao Fang
	Xiebai-Ointment	AMB, *Angelica sinensis*, *Angelica dahurica*, Goat spinal cord	Muscle growth and pain relief	Beiji Qianjin Yao Fang
	AMB	AMB	Muscle production, fetus settling, heartache	Qianjin Yi Fang
	AMB	AMB	Weapon damage	Xinxiu Bencao
	AMB	AMB	Stroke	Shiliao Bencao
	Chi-Xie-Decoction	AMB, Fermented black beans	Typhoid fever, abdominal pain	Waitai Miyao
	Bu-Wei-Decoction	AMB, Poria cocos, *Panax ginseng*, Pericarpium citri reticulatae, *Zingiber officinale*, Fermented black beans, Polished glutinous rice	Stomach maintenance	Waitai Miyao
	Chen-Tong-Powder	AMB, *Achyranthes bidentata*, *Angelica sinensis*, *Cinnamomum cassia*, *Atractylodes macrocephala*, *Astragalus membranaceus*, Radix angelicae tuhuo, *Zingiber officinale*, *Glycyrrhiza uralensis*	Benefiting Qi, tonifying blood, warming menstruation and relieving pain	Jingxiao Chanbao
Song Dynasty	Huanglian-Decoction	AMB, Coptis chinensis, *Gardenia jasminoides* (nuts), Fermented black beans	Dysentery	Taiping Shenghui Fang
	Xiebai-Renshen-Powder	AMB, *Panax ginseng*, *Atractylodes macrocephala*, *Houpoea officinalis*, *Elsholtzia ciliata*	Cholera, dry heaving	Taiping Shenghui Fang
	Xiebai-Decoction	AMB, *Glycyrrhiza uralensis*, *Angelica sinensis*, *Sanguisorba officinalis*, Polished glutinous rice	Dysentery with abdominal pain in pregnancy	Taiping Shenghui Fang
	Jiao-Chi-Decoction	AMB, Collacoriiasini, Fermented black beans, *Zingiber officinale*	Postpartum cold and dysentery, diarrhea and abdominal pain	Taiping Shenghui Fang
	AMB	AMB	Tonic for deficiency and detoxification	Bencao Tujing
	Xiebai-Decoction	AMB, Fermented black beans, *Gardenia jasminoides* (nuts)	Typhoid fever, abdominal pain	Leizheng Huoren Shu
	AMB	AMB	Burn and scald	Bencao Yanyi
	Cong-Xie-Decoction	AMB, *A. fistulosum* (white part), Schizonepeta spike, Caulis bambusae, Fermented black beans, *Zingiber officinale*, Bunge pricklyash seed	Typhoid fever	Sheng Ji Zonglu
	Huangqi-Xiebai-Decoction	AMB, *Panax ginseng*, Poria cocos (white part), *Schisandra chinensis*, *Atractylodes macrocephala*, *A. fistulosum* (white part), Polished glutinous rice, *Paeonia lactiflora* (white), *Zingiber officinale*, Goat or Sheep kidney	Weakness after typhoid fever	Sheng Ji Zonglu
	Congbai-Decoction	AMB, *A. fistulosum* (white part), *Glycyrrhiza uralensis*, Artemisia apiacea, Almond	Night sweats, muscle wasting	Sheng Ji Zonglu
	Shexiang-Decoction	AMB, Bupleurum fruticosum, Ferulae resina, *Glycyrrhiza uralensis*, Artemisia apiacea, Semen persicae, Willow branch, *Rosa laevigata* (branch), *A. fistulosum* (white part), *Areca catechu*	Tuberculosis	Sheng Ji Zonglu
	Xiebai-Noodles	AMB, *Zingiber officinale*, Flour	Post-typhoid dysentery with water and grain	Sheng Ji Zonglu
	Goji-Berry-Porridge	AMB, Goji Berry, *A. fistulosum* (white part), Fermented black beans, Rice	Weakness after typhoid fever and pain in the back	Sheng Ji Zonglu
	Ejiao-Pieces	AMB, Collacoriiasini, Dried ginger	Dysentery	Sheng Ji Zonglu
	Xiebai-Cake	AMB, Egg yolk, Amber	Watery dysentery, dysentery with purulent and bloody stools	Sheng Ji Zonglu
	La-Xie-Cake	AMB, Paraffin, Egg, Flour	Dysentery with purulent and bloody stools	Sheng Ji Zonglu
Yuan Dynasty	AMB	AMB	Dysentery	Tangye Becao
	AMB	AMB	Long-term dysentery, cholera	Bencao Yuanming Bao
Ming Dynasty	Xiebai-Powder	AMB, *Trionyx sinensis*, Collacoriiasini, Antler glue	Prolonged cough, vomiting of blood, hemoptysis	Qixiao Liangfang
	Baishuji-Porridge	AMB, *Tremella fuciformis*, Rice	Dysentery with purulent and bloody stools	Yifang Leiju
	AMB	AMB	Thoracic obstruction and tingling, calming the fetus	Bencao Gangmu
	Xiebai-Chen-Tong-Powder	AMB, *Astragalus membranaceus*, *Angelica sinensis*, Achyranthes bidentata, *Cinnamomum cassia*, *Atractylodes macrocephala*, Radix angelicae tuhuo, *Zingiber officinale*, *Glycyrrhiza uralensis*	Postpartum weakness and pain around the body	Chishui Xuanzhu
	AMB	AMB	Warming the stomach and removing food stagnation	Bencao Huiyan
Qing Dynasty	AMB	AMB	Cough and asthma	Bencao Beiyao
	AMB	AMB	Promoting muscle production, dispersing nodules, relieving asthma and calming the fetus	Bencao Yidu
	AMB	AMB	Food accumulation, worm accumulation	Benjing Fengyuan
	AMB	AMB	Relieving diarrhea, calming the fetus and relieving pain	Cahngsha Yaojie
	AMB	AMB	Giving birth, muscle and dysentery	Bencao Congxin
	AMB	AMB	Relieving diarrhea, promoting blood circulation, relieving asthma, relieving pain and calming the fetus	Bencao Qiuzhen
	AMB	AMB	Cardiothoracic pain, back pain	Yao Zheng
	Leng-Xie-Duan-Lou-Pills	AMB, Arcae concha, Chicken’s Gizzard-membrane, *Corydalis yanhusuo*, Myrrh, *Cyperus rotundus*, Semen persicae, *Trichosanthes kirilowii* (nuts), *Perilla frutescens* (seeds), *Sinapis alba* (seeds), *Raphanus sativus* (seeds)	Abdominal mass, stagnation of phlegm and dyspepsia	Yiji Baojian
	AMB	AMB	Invigorates the muscles, moves Qi and invigorates blood	Bencao Fenjing
	AMB	AMB	Stroke and CHD	Yaoxing Jiyao Bianlan
	AMB	AMB	Dispersing nodules, relieving pain, relieving diarrhea and calming the fetus	Suixi Juyin Shipu
	JiaWei-Baihe-Decoction	AMB, *Lindera aggregata*, Lilii Bulbus, Fritillary, *Trichosanthes kirilowii*, Cardamom	Chest and diaphragm pain	Buzhi Yi Biyao
	AMB	AMB	Promoting Qi flow and stopping diarrhea	Benbao Biandu
	JiaWei-Si-Ni-Powder	AMB, Bupleurum fruticosum, *Citrus aurantium*, *Paeonia lactiflora* (white), Dried ginger, *Glycyrrhiza uralensis* (fried with honey), Cassia twig, Poria cocos, Radix aconiti lateralis preparata	Deadly cold hand and foot, dry cough, palpitations, abdominal pain	Chongding Tongsu Shanghan Lun
Modern	Qingyi-Pills	AMB, Bupleurum fruticosum, *Scutellaria baicalensis*, *Pinellia ternata*, *Trichosanthes kirilowii*, *Citrus aurantium*, Szechwan chinaberry fruit, *Paeonia lactiflora* (white), Chinese rhubarb	Abdominal pain, hypochondriac pain, and back pain in the recovery period of acute pancreatitis	New Acute Abdominology
	Xinnaoning-Capsules	AMB, Ginkgo leaves, Buxus microphylla, *Salvia miltiorrhiza*, *Litsea lancilimba*	CHD, cerebral arteriosclerosis	ChP (2020)
	Xuezhitong-Capsules	AMB	Hyperlipidemia	ChP (2020)
	Dan-Lou-Tablets	AMB, *Trichosanthes kirilowii*, *Salvia miltiorrhiza*, Radix puerariae, *Ligusticum chuanxiong*, *Paeonia lactiflora* (red), *Alisma plantago-aquatica*, *Astragalus membranaceus*, *Davallia mariesii*, Radix curcumae	CHD, AP	ChP (2020)
	Tongxiening-Granules	AMB, *Paeonia lactiflora* (white), Pericarpium citri reticulatae viride, *Atractylodes macrocephala*	Abdominal pain, diarrhea	ChP (2020)
	Buxinqi-Oral Liquid	AMB, *Astragalus membranaceus*, *Panax ginseng*, *Acorus tatarinowii*	Thoracic obstruction and heartache	ChP (2020)
	Zhenxintong-Oral Liquid	AMB, *Codonopsis pilosula*, *Panax notoginseng*, *Corydalis yanhusuo*, Earthworm, Semen lepidii, *Cinnamomum cassia*, Borneol, Menthol	CHD, AP	ChP (2020)

**Table 4:** List of steroids and steroidal saponins isolated from AMB.

Classification	No.	Skeleton	Ingredient Name	R_1_	R_2_	R_3_	R_4_	R_5_	R_6_	Sources	Reference
Spirostanol saponins	**1**	A1	Macrostemonoside A	Gal(1-4)-Glc-[(1-2)-Glc]-(1-3)-Glc	H	H	H	-	-	*A. macrostemon A. chinense*	[8,9]
	**2**		Macrostemonoside D	Gal(1-4)-Glc-[(1-2)-Glc-(1-6)-Ac]-(1-3)-Glc	H	H	H	-	-	*A. macrostemon A. chinense*	[8,10]
	**3**		(3β,5β,12β,25R)-12-hydroxyspirostan-3-yl-2-O-β-D-glucopyranosyl-β-D-galactopyranoside	Gal(1-2)-Glc	H	OH	H	-	-	*A. macrostemon*	[11]
	**4**		(2β,3β,5β,25R)-2-hydroxyspirostan-3-yl-2-O-β-D-glucopyranosyl-β-D-galactopyranoside	Gal(1-2)-Glc	OH	H	H	-	-	*A. macrostemon*	[12]
	**5**		Timosaponin AII	Gal(1-2)-Glc	OH	H	H	-	-	*A. macrostemon*	[12]
	**6**		Schidigera saponin C2	Gal(1-2)-Glc	OH	H	H	-	-	*A. macrostemon*	[12]
	**7**		(3β, 5β, 25R)-spirostan-3-yl-2-O-β-D-glucopyranosyl-β-D-galactopyranoside	Gal(1-2)-Glc	H	H	H	-	-	*A. macrostemon*	[13]
	**8**		Smilagenin	H	H	H	H	-	-	*A. macrostemon*	[13]
	**9**		Laxogenin	H	H	H	O	-	-	*A. macrostemon A. chinense*	[12,14]
	**10**		Xiebai saponin I	Glc[(1-4)-Xyl]-(1-6)-Ara	H	H	O	-	-	*A. macrostemon A. chinense*	[9,12]
	**11**		Smilaxin A	Glc-(1-6)-Ara	H	H	O			*A. macrostemon A. chinense*	[12,14]
	**12**		(3β,5β)-spirost-25(27)-en-3-yl-2-O-β-D-glucopyranosyl-β-D-galactopyranoside	Gal(1-2)-Glc	H	H	H	-	-	*A. macrostemon*	[11]
	**13**		Odospiroside	Gal(1-4)-Glc-[(1-2)-Glc]-(1-3)-Glc	H	H	H	-	-	*A. macrostemon*	[11]
	**14**		(25R)-spirostane-5(6)-en-3β-3-O-β-D-glucopyranosyl(1→2)[β-D-glucopyranosyl(l→3)]-β-D-glucopyranosyl-6-acetyl(l→4)-β-D-galactopyranoside	Gal(1-4)-Glc-[(1-2)-Glc-(1-6)-Ac]-(1-3)-Glc	H	H	H	-	-	*A. macrostemon*	[11]
	**15**		Macrostemonoside S	Gal(1-2)-Glc	H	OH	H	-	-	*A. macrostemon*	[11]
	**16**		(2α, 3β, 5α, 25S)-2-hydroxyspirostan-3-yl-O-β-D-glucopyranosyl-(1→2)-O-[β-D- glucopyranosyl-(1→3)]-O-β-D-glucopyranosyl-(1→4)-β-D-galactopyranoside	Gal(1-4)-Glc-[(1-2)-Glc]-(1-3)-Glc	OH	H	H	-	-	*A. chinense*	[15]
	**17**		(2α, 3β, 5α, 25R)-2-hydroxyspirostan-3-yl-O-β-D-glucopyranosyl-(1→2)-O-[β-D- glucopyranosyl-(1→3)]-O-β-D-glucopyranosyl-(1→4)-β-D-galactopyranoside	Gal(1-4)-Glc-[(1-2)-Glc]-(1-3)-Glc	OH	H	H	-	-	*A. chinense*	[15]
	**18**		(2α, 3β, 5α, 25S)-2-hydroxyspirostan-3-yl-O-β-D-glucopyranosyl-(1→2)-O-β-D- glucopyranosyl- (1→4)-β-D-galactopyranoside	Gal(1-4)-Glc-(1-2)-Glc	OH	H	H	-	-	*A. chinense*	[15]
	**19**		Petunioside	Gal(1-4)-Glc-(1-2)-Glc	OH	H	H	-	-	*A. chinense*	[15]
	**20**	A2	5β-spirostane-25(27)-en-3β,12β-diol-3-O-β-D-glucopyranosyl-(1→2)-β-D-galactopyranoside	Gal(1-2)-Glc	-	-	-	-	-	*A. macrostemon*	[11]
	**21**	A3	(25R)-5β-spirostane-3β,12β-diol-3-O-β-D-glucopyranosyl-(1→2)-β-D-galactopyranoside	Gal(1-2)-Glc	-	-	-	-	-	*A. macrostemon*	[11]
	**22**	A4	5β-spirostane-25(27)-en-2β,3β-diol-3-O-β-D-glucopyranosyl(1→2)-β-D-galactopyranoside	Gal(1-2)-Glc	-	-	-	-	-	*A. macrostemon*	[11]
	**23**	A5	5β-spirostane-25(27)-en-3β-3-O-β-D-glucopyranosyl-(1→2)-β-D-galactopyranoside	Gal(1-2)-Glc	-	-	-	-	-	*A. macrostemon*	[11]
	**24**	A6	Odospiroside	Gal(1-4)-Glc-[(1-2)-Glc]-(1-3)-Glc	-	-	-	-	-	*A. macrostemon*	[11]
	**25**	A7	Chinenoside VI	Glc(1-6)-Ara	Glc	-	-	-	-	*A. chinense*	[16]
	**26**	A8	Allimacrosides B	Gal(1-4)-Glc-[(1-2)-Glc]-(1-3)-Glc	Glc	-	-	-	-	*A. macrostemon*	[17]
	**27**	A9	Allimacrosides C	Gal(1-4)-Glc-[(1-2)-Glc]-(1-3)-Glc	Glc	-	-	-	-	*A. macrostemon*	[17]
	**28**	A10	(25R,S)-26-O-β-D-glucopyranosyl-5α-spirotane-3β-ol-3-O-β-D-glucopyranosyl-(1→2)-[β-Dglucopyranosyl-(1→3)]-(6-acetyl-β-D-glucopyranosyl-(1→4)-β-D-galacopyranosid	Gal(1-4)-Glc-6-acetyl-[(1-2)-Glc]-(1-3)-Glc	-	-	-	-	-	*A. chinense*	[18]
Furostanol saponins	**29**	B1	Macrostemonoside B	Gal(1-4)-Glc-[(1-2)-Glc]-(1-3)-Glc	H	H	H	H	H	*A. macrostemon A. chinense*	[18,19]
	**30**		Macrostemonoside C	Gal(1-4)-Glc-[(1-2)-Glc]-(1-3)-Glc	H	H	H	CH_3_	H	*A. macrostemon*	[19]
	**31**		Macrostemonoside G	Gal(1-2)-Glc	H	H	OH	H	H	*A. macrostemon*	[19]
	**32**		Macrostemonoside H	Gal(1-2)-Glc	H	H	OH	CH_3_	H	*A. macrostemon*	[20]
	**33**		Macrostemonoside I	Gal(1-2)-Glc	H	H	OH	H	H	*A. macrostemon*	[20]
	**34**		Macrostemonoside J	Gal(1-2)-Glc	OH	H	H	H	H	*A. macrostemon*	[21]
	**35**		Macrostemonoside K	Gal(1-2)-Glc	OH	H	H	CH_3_	H	*A. macrostemon*	[22]
	**36**		Macrostemonoside M	H	OH	OH	H	H	OH	*A. macrostemon*	[19]
	**37**		Macrostemonoside N	H	OH	OH	H	H	OH	*A. macrostemon*	[19]
	**38**		Macrostemonoside O	Gal(1-2)-Glc	H	H	H	H	H	*A. macrostemon*	[21]
	**39**		Macrostemonoside P	Gal(1-2)-Glc	H	OH	H	H	H	*A. macrostemon*	[21]
	**40**		Macrostemonoside Q	Gal(1-2)-Glc	OH	OH	H	H	H	*A. macrostemon*	[21]
	**41**		Macrostemonoside R	Gal(1-4)-Glc-[(1-2)-Glc]-(1-3)-Glc	OH	H	H	H	H	*A. macrostemon*	[21]
	**42**		(3β,5α,12β,25R)-26-O-β-D-glucopyranosyloxy-12,22-dihydroxyfurostan-3-yl-O-β-D-glucopyranosyl-(1→2)-O-[β-D-glucopyranosyl-(1→3)]-O-β-D-glucopyranosyl-(1→4)-β-D-galactopyranoside	Gal(1-4)-Glc-[(1-2)-Glc]-(1-3)-Glc	H	H	OH	H	H	*A. macrostemon*	[19]
	**43**		(3β,5α,12β)-26-O-β-D-glucopyranosyloxy-12,22-dihydroxyfurost-25-en-3-yl-O-β-D-glucopyranosyl-(1→2)-O-[β-D-glucopyranosyl-(1→3)]-O-β-D-glucopyranosyl-(1→4)-β-D-galactopyranoside	Gal(1-4)-Glc-[(1-2)-Glc]-(1-3)-Glc	H	H	OH	H	H	*A. macrostemon*	[19]
	**44**		(3β,5α,12α,25R)-26-O-β-D-glucopyranosyloxy-12,22-dihydroxyfurostan-3-yl-O-β-D-glucopyranosyl-(1→2)-O-[β-D-glucopyranosyl-(1→3)]-O-β-D-glucopyranosyl-(1→4)-β-D-galactopyranoside	Gal(1-4)-Glc-[(1-2)-Glc]-(1-3)-Glc	H	H	OH	H	H	*A. macrostemon*	[23]
	**45**		(3β,5β,12α,25R)-26-O-β-D-glucopyranosyloxy-12,22-dihydroxyfurostan-3-yl-2-O-β-D-glucopyranosyl-β-D-galactopyranoside	Gal(1-2)-Glc	H	H	OH	H	H	*A. macrostemon*	[23]
	**46**		Elephanoside E	Gal(1-2)-Glc	H	H	OH	H	H	*A. macrostemon*	[23]
	**47**		(3β,5β,12β,25R)-26-O-β-D-glucopyranosyloxy-22-methoxy-12-hydroxyfurostan-3-yl-2-O-β-D-glucopyranosyl-β-D-galactopyranoside	Gal(1-2)-Glc	H	H	OH	CH_3_	H	*A. macrostemon*	[19]
	**48**		(3β,5β,12α,25R)-26-O-β-D-glucopyranosyloxy-22-methoxy-12-hydroxyfurostan-3-yl-2-O-β-D-glucopyranosyl-β-D-galactopyranoside	Gal(1-2)-Glc	H	H	OH	CH_3_	H	*A. macrostemon*	[19]
	**49**		(3β,5β)-26-O-β-D-glucopyranosyloxy-22-methoxy-25(27)-en-12-onefurost-3-yl-2-O-β-D-glucopyranosyl-β-D-galactopyranoside	Gal(1-2)-Glc	H	H	OH	CH_3_	H	*A. macrostemon*	[19]
	**50**		(1β,3β,5β,6β,22α)-26-O-β-D-glucopyranosyloxy-1,6,22-trihydroxyfurost-25-en-3-yl-β-D-galactopyranoside	Gal	H	OH	H	H	H	*A. macrostemon*	[24]
	**51**		Timosaponin B II	Gal(1-2)-Glc	H	H	H	H	H	*A. macrostemon*	[11]
	**52**		(25R)-26-O-β-D-glucopyranosyl-22-hydroxy-5β-furost-3β,26-diol-3-O-β-D-glucopyranosyl-(1→2)-β-D-galactopyranoside	Gal(1-2)-Glc	H	H	H	H	H	*A. macrostemon*	[11]
	**53**		(3β,25R)-26-O-β-D-glucopyranosyloxy-22-hydroxyfurost-5-en-3-yl-O-β-D-glucopyranosyl-(1→2)-O-[β-D-glucopyranosyl-(1→3)]-O-β-D-glucopyranosyl-(1→4)-β-D-galactopyranoside	Gal(1-4)-Glc-[(1-2)-Glc]-(1-3)-Glc	H	H	H	H	H	*A. macrostemon*	[25]
	**54**		Chinenoside I	Glc[(1-4)-Xyl]-(1-6)-Ara	H	H	H	H	O	*A. chinense*	[7]
	**55**	B2	Macrostemonoside E	Gal(1-4)-Glc-[(1-2)-Glc]-(1-3)-Glc	H	H	H	H	-	*A. macrostemon*	[19]
	**56**		Macrostemonoside F	Gal(1-2)-Glc	H	H	H	H	-	*A. macrostemon*	[19]
	**57**		Macrostemonoside L	Gal(1-2)-Glc	OH	H	H	H	-	*A. macrostemon*	[22]
	**58**		(3β,5β,12β)-26-O-β-D-glucopyranosyloxy-5β-furost-20(22)-25(27)-dien-3β,12β,26-triol-3-β-2-O-β-D-glucopyranosyl-β-D-galactopyranoside	Gal(1-2)-Glc	H	H	OH	H	-	*A. macrostemon*	[19]
	**59**		(3β,5β,12α,25R)-26-O-β-D-glucopyranosyloxy-12-hydroxyfurost-20(22)-en-3-yl-2-O-β-D-glucopyranosyl-β-D-galactopyranoside	Gal(1-2)-Glc	H	H	OH	H	-	*A. macrostemon*	[19]
	**60**		Chinenoside II	Glc-[(1-4)-Xyl]-(1-6)-Ara	H	H	H	O	-	*A. chinense*	[26]
	**61**		Chinenoside III	Glc-(1-6)-Ara	H	H	H	O	-	*A. chinense*	[26]
	**62**		26-O-β-D-glucopyranosyl-5β-furostane-20(22)-25(27)-dien-3β,26-diol-3-O-β-D-glucopyranosyl-(l→2)-β-D-galactopyranoside	Gal(1-2)-Glc	H	H	H	H	-	*A. macrostemon*	[11]
	**63**	B3	(25R)-26-O-β-D-glucopyranosyl-22-hydroxy-furost-5(6)-ene-3β,26-diol-3-O-β-D-glucopyranosyl(1→2)[β-D-glucopyranosyl(1→3)]-β-D-glucopyranosyl(1 →4)-β-D-galactopyranoside	Gal(1-4)-Glc-[(1-2)-Glc]-(1-3)-Glc	Gal	-	-	-	-	*A. macrostemon*	[25]
	**64**	B4	(25R)-26-O-β-D-glucopyranosyl-5α-furostane-3β,12β,22,26-tetraol-3-O-β-D-glucopyranosyl(1→2)[β-D-glucopyranosyl(1→3)]-β-D-glucopyranosyl (1→4)-β-D-galactopyranoside	Gal(1-4)-Glc-[(1-2)-Glc]-(1-3)-Glc	β-OH	-	-	-	-	*A. macrostemon A. chinense*	[18,27]
	**65**		(25R)-26-O-β-D-glucopyranosyl-5α-furostane-3β,12α,22,26-tetraol-3-O-β-D-glucopyranosyl (1→2) [β-D-glucopyranosyl (1→3)]-β-D-glucopyranosyl (1→4)-β-D-galacto- pyranoside	Gal(1-4)-Glc-[(1-2)-Glc]-(1-3)-Glc	α-OH	-	-	-	-	*A. macrostemon*	[27]
	**66**	B5	(25R)-26-O-β-D-glucopyranosyl-5β-furostane-3β,12α,22,26-tetraol-3-O-β-D-glucopyranosyl (1→2)-β-D-galactopyranoside	Glc(1-2)-Glc	OH	-	-	-	-	*A. macrostemon*	[27]
	**67**	B6	(25R)-26-O-β-D-glucopyranosyl-5β-furostane-12β,3β,22,26-tetraol-3-O-β-D-glucopyranosyl (1→2)-β-D-galactopyranoside	Glc(1-2)-Glc	OH	-	-	-	-	*A. macrostemon*	[23]
	**68**	B7	(25R)-26-O-β-D-glucopyranosyl-5β-furostane-22(23)-en-20-methoxyl-3β,26-diol-3-O-β-D-glucopyranosyl (1→2)-β-D-galactopyranoside	Gal(1-2)-Glc	-	-	-	-	-	*A. macrostemon*	[11]
	**69**	B8	(25R)-26-O-β-D-glucopyranosyl-5β-furostane-20(22)-en-3β,12α,26-triol-3-O-β-D-glucopyranosyl(l→2)-β-D-galactopyranoside	Gal(1-2)-Glc	-	-	-	-	-	*A. macrostemon*	[11]
	**70**	B9	(25S)-26-O-β-D-glucopyranosyl-5α-furostane-2α,3β,22,26-tetraol-3-O-β-D-glucopyranosyl-(1→2)-[β-D-glucopyranosyl-(1→3)]-β-D-glucopyranosyl(1→4)-β-D-galactopyranoside	Gal(1-4)-Glc-[(1-2)-Glc]-(1-3)-Glc	Gal	-	-	-	-	*A. macrostemon*	[28]
	**71**	B10	25(27)-ene-26-O-β-D-glucopyranosyl-5α-furostane-3β,22,26-triol-3-O-β-D-glucopyra-nosyl-(1→2)-[β-D-glucopyranosyl-(1→3)]-β-D-glucopyranosyl(1→4)-β-D-galact opyranoside	Gal(1-4)-Glc-[(1-2)-Glc]-(1-3)-Glc	Gal	-	-	-	-	*A. macrostemon*	[28]
	**72**	B11	Allimacrosides D	Gal(1-4)-Glc-[(1-2)-Glc]-(1-3)-Glc	Glc	-	-	-	-	*A. macrostemon*	[17]
	**73**	B12	Allimacrosides E	Gal(1-4)-Glc-[(1-2)-Glc]-(1-3)-Glc	Glc	-	-	-	-	*A. macrostemon*	[17]
	**74**	B13	Chinenoside IV	Glc-[(1-4)-Xyl]-(1-6)-Ara	-	-	-	-	-	*A. chinense*	[29]
	**75**		Chinenoside V	Glc-(1-6)-Ara	-	-	-	-	-	*A. chinense*	[29]
	**76**		(25R)-6-one-5α-furostane-3β,26-triol-20(22)-en-26-O-β-D-glucopyranoside	H	-	-	-	-	-	*A. chinense*	[30]
	**77**	B14	5α-cholano-22,16-lactone-3-hydroxyl-3-O-β-D-glucopyranosyl-(1→2)-[β-D-glucopyranosyl-(1→3)]-β-D-glucopyranosyl-(1→4)-β-D-galacopyranoside	Gal(1-4)-Glc-[(1-2)-Glc]-(1-3)-Glc	-	-	-	-	-	*A. chinense*	[31]
	**78**	B15	6-one-5α-cholano-22,16-lactone-3-hydroxyl-3-O-β-D-xylopyranosyl-(1→4)-[α-L-arabinopyranosyl-(1→6)]-β-D-glucopyranoside	Glc[(1-4)-Xyl]-(1-6)-Ara	-	-	-	-	-	*A. chinense*	[31]
	**79**	B16	(25R)-26-O-β-D-glucopyranosyl-5α-furostane-3β,26-diol-3-O-β-D-glucopyranosyl-(1→2)-[β-D-glucopyranosyl-(1→3)]-β-D-glucopyranosyl-(1→4)-β-D-galacopyranoside	Gal(1-4)-Glc-[(1-2)-Glc]-(1-3)-Glc	Glc	-	-	-	-	*A. chinense*	[31]
	**80**	B17	(25R)-6-one-26-O-β-D-glucopyranosyl-5α-furostane-3β,22α,26-triol-3-O-β-D-xylopyranosyl-(1→4)-β-D-glucopyranoside	Glc(1-4)-Xyl	Glc	H	-	-	-	*A. chinense*	[31]
	**81**		(25R)-6-one-5α-furostane-3β,22α,24β,26-tetraol-3-O-β-D-xylopyranosyl-(1→4)-[α-L-arabinopyranosyl-(1→6)]-β-D-glucopyranoside	Glc[(1-4)-Xyl]-(1-6)-Ara	H	OH	-	-	-	*A. chinense*	[31]
	**82**		(25R)-6-one-26-O-β-D-glucopyranosyl-5α-furostane-3β,22,26-triol-3-O-α-L-arabinopyranosyl-(1→6)-β-D-glucopyranoside	Glc(1-6)-Ara	Glc	H	-	-	-	*A. chinense*	[18]
	**83**		(25R)-6-one-26-O-β-D-glucopyranosyl-5α-furostane-3β,22,26-triol-3-O-β-D-xylopyranosyl-(1→4)-[α-L-arabinopyranosyl-(1→6)]-β-D-glucopyranoside	Glc[(1-4)-Xyl]-(1-6)-Ara	Glc	H	-	-	-	*A. chinense*	[18]
	**84**		(25R)-6-one-5α-furostane-3β,22α,26-triol-26-O-β-D-glucopyranoside	H	Glc	H	-	-	-	*A. chinense*	[18]
	**85**		(25R)-6-one-26-O-β-D-glucopyranosyl-5α-furostane-3β,22α,26-triol-3-O-β-D-glucopyranoside	Glc	Glc	H	-	-	-	*A. chinense*	[18]
	**86**	B18	(25R)-26-O-β-D-glucopyranosyl-5α-furostane-2α,3β,22,26-tetraol-3-O-β-D-glucopyranosyl-(1→2)-[β-D-glucopyranosyl-(1→3)]-β-D-glucopyranosyl-(1→4)-β-D-galacopyranoside	Gal(1-4)-Glc-[(1-2)-Glc]-(1-3)-Glc	Glc	OH	-	-	-	*A. chinense*	[18]
	**87**		(25R)-5α-furostane-2β,3β,22α,26-tetraol-26-O-β-D-glucopyranoside	H	Glc	OH	-	-	-	*A. chinense*	[31]
	**88**		(25R)-26-O-β-D-glucopyranosyl-5α-furostane-3β,26-didyroxy-3-O-β-D-glucopyranosyl-(1→4)-β-D-galactopyranoside	Gal(1-4)-Glc	Glc	H	-	-	-	*A. chinense*	[30]
	**89**		Tomatoside A	Gal(1-4)-Glc-(1-2)-Glc	Glc	H	-	-	-	*A. chinense*	[30]
Pregnane glycoside	**90**	C	Allimacrosides A	Gal(1-4)-Glc-[(1-2)-Glc]-(1-3)-Glc	-	-	-	-	-	*A. macrostemon*	[17]
Cholestane glycosides	**91**	D1	(1β,3β,16β,22S)-1-[(6-deoxy-α-L-mannopyranosyl)oxy]-3,22-dihydroxycholest-5-en-16-O-β-D-glucopyranoside	Glc	-	-	-	-	-	*A. macrostemon*	[19]
	**92**	D2	(22S)-cholest-5-ene-1β,3β,16β,22-tetraol-1-O-α-L-rhamnopyranosyl-16-O-β-D-glucopyranoside	Rha	Glc	-	-	-	-	*A. macrostemon*	[18]
Sterols	**93**	D3	Sitosterol	-	-	-	-	-	-	*A. macrostemon*	[32]
	**94**	D4	Stigmasterol	-	-	-	-	-	-	*A. macrostemon*	[19]
	**95**	D5	Daucosterol	-	-	-	-	-	-	*A. macrostemon*	[33]
	**96**	D6	Sitosteryl-6’-O-undecane-β-D-glucoside	-	-	-	-	-	-	*A. macrostemon*	[33]

**Table 5:** List of sulfur-containing compounds previously identified from AMB.

Classification	No.	Skeleton	Ingredient Name	R_1_	R_2_	Sources	Reference
Sulfur-containing compounds	**97**	E1	Ethyl *cis*-1-propenyl sulfide	ethyl	*cis*-1-propenyl	*A. chinense*	[36]
	**98**		Diallyl sulfide	allyl	allyl	*A. chinense*	[36]
	**99**		3-[(1-methylethy) thio]-1-propene	isopropyl	allyl	*A. macrostemon*	[37]
	**100**		Methyl allyl sulfide	methyl	allyl	*A. macrostemon*	[38]
	**101**	E2	Methanethiol	-	-	*A. macrostemon*	[39]
	**102**	E3	1-hydroxyl-2-sulfhydryl-ethane	-	-	*A. macrostemon*	[38]
	**103**	E4	2, 4-dimethylthiophene	-	-	*A. macrostemon*	[37]
	**104**	E5	1, 3-dimethylthiophene	-	-	*A. macrostemon*	[38]
	**105**	E6	Dimethyl sulfone	-	-	*A. macrostemon*	[39]
	**106**	E7	2,4-dihydro-4,5-dimethyl-3H-1,2,4-triazole-3-thione	-	-	*A. macrostemon*	[39]
	**107**	E8	3,4-dimethyl-thiophene			*A. macrostemon*	[39]
	**108**	E9	1, 3-propane sultone	-	-	*A. macrostemon*	[40]
	**109**	E10	Isobutyl isothiocyanate	-	-	*A. macrostemon*	[40]
	**110**	E11	1, 3, 2-dioxathiane-2, 2-dioxide	-	-	*A. macrostemon*	[40]
	**111**	F1	Dimethyl disulfide	methyl	methyl	*A. macrostemon A. chinense*	[36,37]
	**112**		Methyl ethyl disulfide	methyl	ethyl	*A. macrostemon A. chinense*	[36,38]
	**113**		Methyl propyl disulfide	methyl	propyl	*A. macrostemon A. chinense*	[36,37]
	**114**		Methyl allyl disulfide	methyl	allyl	*A. macrostemon A. chinense*	[36,37]
	**115**		Methyl *cis*-1-propenyl disulfide	methyl	*cis*-1-propenyl	*A. macrostemon A. chinense*	[36,38]
	**116**		Methyl isopropyl disulfide	methyl	isopropyl	*A. macrostemon*	[38]
	**117**		Methyl butyl disulfide	methyl	butyl	*A. chinense*	[36]
	**118**		Ethyl propyl disulfide	ethyl	propyl	*A. chinense*	[36]
	**119**		Ethyl *cis*-1-propenyl disulfide	ethyl	*cis*-1-propenyl	*A. chinense*	[36]
	**120**		Ethyl *trans*-1-propenyl disulfide	ethyl	*trans*-1-propenyl	*A. chinense*	[36]
	**121**		Propyl propenyl disulfide	propyl	propenyl	*A. macrostemon*	[38]
	**122**		Propyl isopropyl disulfide	propyl	isopropyl	*A. macrostemon*	[37]
	**123**		Propyl allyl disulfide	propyl	allyl	*A. macrostemon A. chinense*	[37,41]
	**124**		Diallyl disulfide	allyl	allyl	*A. macrostemon A. chinense*	[36,38]
	**125**		Allyl isopropyl disulfide	allyl	isopropyl	*A. macrostemon A. chinense*	[37,41]
	**126**		Allyl *cis*-1-propenyl disulfide	allyl	*cis*-1-propenyl	*A. chinense*	[36]
	**127**		Allyl *trans*-1-propenyl disulfide	allyl	*trans*-1-propenyl	*A. chinense*	[36]
	**128**		*bis* (1-methylethyl) disulfide	isopropyl	isopropyl	*A. macrostemon*	[39]
	**129**	F2	1, 3-dimercaptopropane	-	-	*A. macrostemon*	[38]
	**130**	F3	1,3-dithiane	-	-	*A. macrostemon A. chinense*	[37,41]
	**131**	F4	2, 2-bis(methylthio)propane	-	-	*A. macrostemon*	[37]
	**132**	F5	3-mercapto-2-(mercaptomethyl)-propanoic acid	-	-	*A. macrostemon*	[39]
	**133**	F6	2-ethylidene [1,3]dithiane	-	-	*A. macrostemon*	[39]
	**134**	F7	S-methyl methanethiosulfinate	-	-	*A. macrostemon*	[39]
	**135**	G1	Dimethyl trisulfide	methyl	methyl	*A. macrostemon A. chinense*	[36,37]
	**136**		Methyl ethyl trisulfide	methyl	ethyl	*A. chinense*	[36]
	**137**		Methyl butyl trisulfide	methyl	butyl	*A. chinense*	[36]
	**138**		Methyl propyl trisulfide	methyl	propyl	*A. macrostemon A. chinense*	[36,37]
	**139**		Methyl allyl trisulfide	methyl	allyl	*A. macrostemon A. chinense*	[36,37]
	**140**		Methyl *cis*-1-propenyl trisulfide	methyl	*cis*-1-propenyl	*A. chinense*	[36]
	**141**		Methyl *trans*-1-propenyl trisulfide	methyl	*trans*-1-propenyl	*A. macrostemon A. chinense*	[36,37]
	**142**		Dipropyl trisulfide	propyl	propyl	*A. macrostemon*	[37]
	**143**		Propyl allyl trisulfide	propyl	allyl	*A. macrostemon A. chinense*	[37,41]
	**144**		Diallyl trisulfide	allyl	allyl	*A. macrostemon A. chinense*	[38,41]
	**145**	G2	3, 5-dimethyl-1, 2, 4-tridithiane	-	-	*A. macrostemon*	[37]
	**146**	G3	4-methyl-1, 2, 3-tridithiane	-	-	*A. macrostemon*	[37]
	**147**	G4	3,5-diethyl-1,2,4-trithiolane	-	-	*A. macrostemon*	[39]
	**148**	H1	Dimethyl tetrasulfide	methyl	methyl	*A. macrostemon A. chinense*	[36,37]
	**149**		Methyl pentyl tetrasulfide	methyl	pentyl	*A. chinense*	[36]
	**150**		Propyl *cis*-l-propenyl tetrasulfide	propyl	*cis*-l-propenyl	*A. chinense*	[36]
	**151**		Propyl *trans*-l-propenyl tetrasulfide	propyl	*trans*-l-propenyl	*A. chinense*	[36]
	**152**	H2	5-methyl-1, 2, 3, 4-tetradithiane	-	-	*A. macrostemon*	[37]
	**153**	I	Methyl propyl pentasulfide	methyl	propyl	*A. chinense*	[36]
	**154**		Propyl *cis*-l-propenyl pentasulfide	propyl	*cis*-l-propenyl	*A. chinense*	[36]

**Table 6:** List of nitrogen-containing compounds previously identified from AMB.

Classification	No.	Skeleton	Ingredient Name	Sources	Reference
Nitrogen-containing compounds	**155**	J1	Adenosine	*A. macrostemon A. chinense*	[42,47]
	**156**	J2	Thymidine	*A. macrostemon*	[42]
	**157**	J3	Guanosine	*A. chinense*	[45]
	**158**	K1	2,3,4,9-tetrahydro-1H-pyrido[3,4-b]indole-3- carboxylic acid	*A. macrostemon A. chinense*	[42,47]
	**159**	K2	2,3,4,9-tetrahydro-1-methyl-1H-pyrido[3,4-b]indole-3-carboxylic acid	*A. macrostemon*	[42]
	**160**	L1	Tryptophan	*A. macrostemon A. chinense*	[42,47]
	**161**	L2	N-*trans*-feruloyltyramine	*A. chinense*	[43]
	**162**	L3	N-(*p-trans*-coumaroyl)-tyramine	*A. chinense*	[44]
	**163**	L4	N-(*p-cis*-coumaroyl)-tyramine	*A. chinense*	[44]

**Table 7:** List of phenylpropanoid and flavonoid components isolated from AMB.

Classification	No.	Skeleton	Ingredient Name	R_1_	R_2_	R_3_	Sources	Reference
Phenylpropanoids	**164**	M	Acanthoside D	-	-	-	*A. chinense*	[48]
	**165**	N	Syringin	-	-	-	*A. macrostemon*	[42]
	**166**	O	Allimacronoid A	Glc[(1-2)-Glc]-(1-6)-Glc	-	-	*A. macrostemon*	[50]
	**167**		Allimacronoid B	Glc(1-4)-Glc-[(1-2)-Glc]-(1-6)-Glc	-	-	*A. macrostemon*	[50]
	**168**		Allimacronoid C	Glc(1-2)-Glc-[(1-6)-Glc]-(1-6)-Glc	-	-	*A. macrostemon*	[50]
	**169**		Allimacronoid D	Glc-(1-2)-Glc-(1-6)-Glc	-	-	*A. macrostemon*	[49]
	**170**		Tuberonoid A	Glc-(1-2)-Glc	-	-	*A. macrostemon*	[50]
	**171**		1-O-(E)-feruloyl-β--D-gentiobioside	Glc-(1-6)-Glc	-	-	*A. macrostemon*	[49]
	**172**		1-O-(E)-feruloyl-β-D-glucopyranoside	Glc	-	-	*A. macrostemon*	[49]
	**173**		*trans*-Ferulic acid	H	-	-	*A. macrostemon*	[49]
Flavonoids	**174**	P	Kaempferol-3-O-β-D-glucoside	Glc	H	H	*A. macrostemon*	[51]
	**175**		Kaempferol-3,7-O-β-D-diglucoside	Glc	Glc	H	*A. macrostemon*	[51]
	**176**		Kaempferol-3,4’-O-β-D-diglucoside	Glc	H	Glc	*A. macrostemon*	[51]
	**177**	Q	Quercetin-3-O-β-D-glucoside	-	-	-	*A. macrostemon*	[51]
	**178**	R	Isorhamnetin-3-O-β-D-glucoside	-	-	-	*A. macrostemon*	[51]
	**179**	S	Isoliquiritigenin	H	-	-	*A. chinense*	[14]
	**180**		Isoliquiritigenin-4-O-glucoside	Glc	-	-	*A. chinense*	[14]

**Table 8:** List of other compounds isolated from AMB.

Classification	No.	Skeleton	Ingredient Name	R_1_	R_2_	Sources	Reference
Others	**181**	T	(3β, 4α)-olean-12-en-28-oic acid-3-O-β-D-galactopyranosyloxy-23-hydroxy-6-O-β-D-xylopyranosyl-β-D-galactopyranosyl ester	Gal(1-4)-Xyl	Gal	*A. macrostemon*	[56]
	**182**	-	Prostaglandin A1	-	-	*A. macrostemon*	[55]
	**183**	-	Prostaglandin B1	-	-	*A. macrostemon*	[55]
	**184**	-	2-ene-butanol	-	-	*A. chinense*	[41]
	**185**	-	Ethyl acetate	-	-	*A. chinense*	[36]
	**186**	-	Limonene	-	-	*A. chinense*	[36]
	**187**	-	Succinic acid	-	-	*A. macrostemon*	[40]
	**188**	-	Tetradecanoic acid	-	-	*A. macrostemon*	[57]
	**189**	-	Oleic acid	-	-	*A. macrostemon*	[37]
	**190**	-	Palmitoleic acid	-	-	*A. macrostemon*	[37]
	**191**	-	Palmitic acid	-	-	*A. macrostemon*	[37]
	**192**	-	Linoleic acid	-	-	*A. macrostemon*	[37]

**Table 9:** Reported pharmacological activities of AMB extract or isolated compounds.

Pharmacological Effects	Source	Extract/Compounds	In Vivo/In Vitro	Mechanism	Models	Results	Reference
Anti-platelet aggregation effect	*A. macrostemon*	**161, 163**	In vitro	-	ADP induces human platelet aggregation	Compound **161** showed significant inhibition of both first-phase and second-phase platelet aggregation, while compound **163** showed inhibition of first-phase aggregation only	[62]
	*A. macrostemon*	**1**	In vitro	-	ADP-induced platelet aggregation in rabbits	Strong inhibitory effect on platelet aggregation, IC_50_ = 0.065 mmol	[8]
	*A. macrostemon*	**55, 56**	In vitro	-	ADP induces human platelet aggregation	All these compounds strongly inhibited platelet aggregation, with IC_50_ = 0.417 mmol for compound **55** and IC_50_ = 0.020 mmol for compound **56**	[13]
	*A. macrostemon A. chinense*	**139**	In vitro	-	-	Strong inhibitory effect on platelet aggregation	[37,41]
	*A. macrostemon* *A. chinense*	**155, 158**	In vitro	-	-	All these compounds strongly inhibited platelet aggregation, with IC_50_ = 0.085 mmol for compound **155** and IC_50_ = 0.188 mmol for compound **158**	[42]
	*A. chinense*	**60, 61**	In vitro	-	ADP induces human platelet aggregation	Compounds **60** and **61** both prolong clotting time	[159]
	*A. macrostemon*	**10, 11**	In vitro	-	ADP or PAF induced platelet aggregation in rabbits	All these compounds strongly inhibited platelet aggregation, with IC_50_ = 0.078 mmol for compound **10** and IC_50_ = 0.082 mmol for compound **11**	[12]
	*A. macrostemon*	**31**	In vitro	-	ADP or PAF induced platelet aggregation in rabbits	Strong inhibitory effect on platelet aggregation, IC_50_ = 0.410 mmol	[19]
	*A. macrostemon*	**64, 65**	In vitro	Inhibition of platelet CD40L expression	ADP-induced platelet activation in rats	All these compounds were able to significantly inhibit the expression of platelet CD40L	[160]
	*A. macrostemon*	**59, 64, 65**	In vitro and in vivo	Inhibition of platelet CD40L expression	ADP-induced adhesion between human platelets and neutrophils	All of these compounds showed significant inhibition of platelet CD40L expression at a concentration of 320 μmol/L. Compound **64** at a concentration of 80 μmol/L and compounds **59** and **65** at a concentration of 320 μmol/L significantly inhibited the adhesion between platelets and neutrophils	[23]
	*A. macrostemon*	**64**	In vitro	-	ADP induces human platelet aggregation	Significantly inhibited platelet aggregation and the expression of P-selectin and integrin β-3, significantly reduced the expression of p-Akt in platelets, and inhibited calcium ion mobilization	[27]
	*A. macrostemon*	AMB saponins	In vitro and in vivo	-	AA, ADF and PAF induced platelet aggregation in rats	Inhibits platelet aggregation and reduces the concentration of calcium ions in washed platelets and adhesion between neutrophils and thrombin-activated platelets, and inhibits platelet aggregation induced by neutrophil supernatant	[61]
	*A. macrostemon*	AMB saponins	In vitro	May be related to CD40L/JNK/P38/NF-κB inflammation-related signaling pathway	ADP induces an inflammatory response in human platelet-derived extracellular vesicles	Inhibits ADP-induced inflammatory response in platelet-derived extracellular vesicles and suppresses inflammatory response in endothelial cells	[161]
	*A. macrostemon*	AMB saponins	In vitro	May act on two ADP receptors P2Y_1_ and P2Y_12_ on platelet membrane to reduce intracytoplasmic calcium ion concentration and increase CAMP content	ADP induces human platelet aggregation	AMB saponin at medium to high doses significantly inhibited platelet aggregation, and AMB saponin at 4 μmol/L significantly reduced the expression rate of CD62p in activated platelets, and the expression rate of GPIIb/IIIa was lower than that after activation	[162]
	*A. macrostemon*	AMB saponins	In vivo	Inhibition of platelet CD40L expression	Establishment of a rat model of coronary heart disease by high-fat diet feeding and injection of posterior pituitary hormone	It can inhibit platelet aggregation, prolong prothrombin time, and thrombin time, activate partial thromboplastin time and reduce plasma fibrinogen content in the arterial blood of rats	[163]
	*A. macrostemon*	**14, 62, 64, 65, 66, 69**	In vitro and in vivo	Inhibition of platelet PI3K expression and Akt phosphorylation	ADP-induced platelet aggregation in rats	All of these compounds inhibit platelet aggregation and inhibit the expansion of platelets on immobilized fibrinogen	[63]
Hypolipidemic and anti-atherosclerotic effects	*A. macrostemon*	AMB 95% ethanol extracts	In vivo	Promotes the secretion of PGE1	Domestic rabbits	Can increase the synthesis of PGE1 in rabbits, thus inhibiting the synthesis of TXA_2_, and can inhibit the formation of experimental atheromatous plaques	[74]
	*A. macrostemon*	AMB aqueous extracts	In vivo	-	High-fat diet and methylthioxypyrimethane-induced hyperlipidemia in rats	Significantly reduced serum levels of TC, TG, and LDL in rats, and reduced atherosclerotic index	[164]
	*A. macrostemon*	**1**	In vitro	Increased visfatin mRNA levels in 3T3-L1 cells and mediated through P38 MAPK	3T3-L1 cells	Compound **1** increases visfatin mRNA levels in 3T3-L1 adipocytes and significantly enhances visfatin protein expression, partly mediated by the MAPK signaling pathway	[76]
	*A. macrostemon*	**1**	In vivo	Increased total lipase activity in visceral adipocytes	High-fat diet-induced hyperglycemia and hyperlipidemia in C57BL/6 mice	Compound **1** significantly reduced serum levels of TC, TG, and LDL, and lowered blood glucose levels in mice	[77]
	*A. chinense*	AMB saponins	In vivo	-	Construction of hyperlipidemic rat model by high-fat diet feeding	It significantly reduced the levels of TC, TG, LDL, and MDA, and significantly increased the levels of HDL, GSH-Px, and SOD in the serum of rats. At the same time, the levels of LPL and HTGL in rat liver were also significantly increased and the production of fat droplets was significantly reduced	[71]
	*A. chinense*	AMB volatile oils	In vivo	-	Construction of hyperlipidemic rat model by high-fat diet feeding	Significantly reduced TC, TG, and LDL levels in serum and liver, and increased HDL levels in serum in rats, in addition to showing protective effects associated with histopathological changes in the liver	[72]
	*A. macrostemon*	10% AMB powder	In vivo	Up-regulation of LDLR, LXRα mRNA expression levels in liver tissues	Construction of hyperlipidemic rat model by high-fat diet feeding	Significantly lowered serum TC and LDL levels and significantly increased serum HDL levels in rats	[75]
	XZT	AMB extracts	In vivo	Activation of RCT and increase in HDL levels	ApoE^−/−^ mices	Significantly reduced the serum levels of FAS and LDL in mice	[79]
	XZT	AMB extracts	In vivo	-	Patients with hyperlipidemia	Significantly reduced TG levels in hyperlipidemic patients	[78]
Protection of cardiomyocytes and vascular endothelial cells	*A. macrostemon*	AMB 5% ethanol extracts	In vitro	Blockade of calcium channels	Isolated rabbit aortic strips	May exert vasodilatory effects by inhibiting calcium channels activated by high potassium and NA	[165]
	*A. macrostemon*	AMB extracts	In vitro	Reduces myocardial oxygen consumption	ISO-induced normoxia model in mice, acute myocardial ischemia model in rats, and myocardial ischemia-reperfusion model in rats caused by the posterior pituitary hormone	It can prolong the survival time of normoxia in mice, counteract acute myocardial ischemia in rats, and significantly protect myocardial injury caused by ischemia-reperfusion in rats	[166]
	*A. macrostemon*	AMB extracts	In vivo	Improvement of abnormal gene expression profiles in vascular lesions	Establishment of qi stagnation vascular endothelial injury model in rats fed with restraint and high methionine diet	Reduces gene expression of COX-2, COX-1, iNOS, ECE, and eNOS, and increases gene expression of antioxidant SOD, thus protecting vascular endothelium	[85]
	*A. macrostemon*	AMB extracts	In vivo	-	Establishment of qi stagnation vascular endothelial injury model in rats fed with restraint and high methionine diet	Reduces COX-2 and iNOS protein content in rat blood vessels, thereby protecting the endothelium from damage	[86]
	*A. macrostemon*	AMB extracts	In vivo	Regulation of 5-HT receptor expression	Stressed rats using restraint method	It can protect vascular endothelial function by enhancing 5-HT_1D_ mRNA and protein expression, which mediates the diastolic effect and inhibiting 5-HT_2A_ mRNA and protein expression, which mediates the vasoconstrictive effect	[90]
	*A. macrostemon*	AMB extracts	In vivo	Inhibition of endoplasmic reticulum stress	Establishment of qi stagnation vascular endothelial injury model in rats fed with restraint and high methionine diet	It can significantly reduce the plasma ET level, increase the serum NO level and inhibit the expression of GRP78 protein in aortic tissues, thus inhibiting the endoplasmic reticulum stress in model rats to improve their vascular endothelial function	[87]
	*A. macrostemon*	AMB ethanol extracts	In vivo	Branched-chain amino acids such as leucine, isoleucine, valine and threonine protect the heart from myocardial infarction damage	Open-chest ligation of the anterior descending branch of the left coronary artery in rats	It can regulate the balance of lipid and protein metabolism and reduce the damage caused by acute myocardial ischemia in the rat organism	[88]
	*A. macrostemon*	AMB extracts	In vivo	-	Open-chest ligation of the anterior descending branch of the left coronary artery in rats	It can increase serum GSH-Px activity, decrease TChE activity, NEFA and MDA content, and reduce the extent of myocardial damage in rats	[89]
Anti-cancer effect	*A. macrostemon*	AMB methanol extracts	In vitro	Associated with its regulation of the EGFR/PI3K/m TOR and RAF/MAPK signaling pathways	Human non-small cell lung cancer A549 and human lung cancer cells PC-9	Ability to significantly inhibit the proliferation of A549 and PC-9	[13]
	*A. chinense*	AMB 20% ethanol extracts	In vivo	-	Tetradecanoyl phorbol acetate (TPA) and dihydroxy methyl butyric acid induced skin cancer model and 5% glycerol and 4-Nitroquinoline-1-oxide (4NQO) induced lung cancer model in mice	It can significantly inhibit the activity of cancer cells in two models of mice	[167]
	*A. chinense*	**25, 60**	In vitro	-	-	All of these compounds have antitumor activity	[168]
	*A. chinense*	**9, 10, 11**	In vitro	Inhibition of TPA-induced phospholipid synthesis in Hela cell membranes	TPA-stimulated ^32^Pi-incorporation into phospholipids of HeLa cells	All of these compounds inhibited Hela cell proliferation, and in addition, compound **9** showed strong inhibitory activity against lung tumor formation induced by both 4-NQO and glycerol in an in vitro lung cancer stage 2 carcinogenesis assay	[14]
	*A. macrostemon*	AMB volatile oils	In vitro and in vivo	Enhance the immune function of tumor-bearing mice, especially the cellular immune function, which is the dominant part of tumor immunity	Mice xenograft model inoculated with mice sarcoma cells S180	It can significantly inhibit tumor growth and increase splenic index, macrophage phagocytosis rate, and splenocyte proliferation index	[93]
	*A. macrostemon*	AMB volatile oils	In vitro and in vivo	Directly kill tumor cells by destroying nucleus and organelles, and promote the expression of cellular wtp53 gene mRNA	A mice xenograft model inoculated with mice sarcoma cells S180 and mice liver cancer cells H22	Inhibits both S180 and H22 in vitro and in vivo, directly kills tumor cells, and induces apoptosis	[94]
	*A. chinense*	AMB extracts	In vitro	Altering the G_2_/M cell cycle of tumor cells	Human hepatocellular carcinoma cells HepG2 and human cervical carcinoma HeLa cells	Strong inhibitory activity against HepG2 and HeLa cells	[97]
	*A. macrostemon*	**30, 52, 63**	In vitro	-	Human neural carcinoma cells SF-268 and human large cell lung cancer cells NCI-H460	These compounds showed good inhibition of SF-268 and NCI-H460 cell growth at 25 mg·L^−1^ mass concentration	[25]
	*A. macrostemon*	AMB volatile oils	In vitro	Promote the expression of P53 protein	Human gastric cancer cells SGC-7901	Able to increase the expression of p53 protein and thus induce apoptosis in SGC-7901 cells	[95]
	*A. macrostemon*	**34, 38, 40, 52**	In vitro	-	Human neural carcinoma cell SF-268, human large cell lung cancer cell NCI-H460, human breast cancer MCF-7, human liver cancer cell HepG2	Compounds **38** and **52** showed significant cytotoxic effects on SF-268, NCI-H460, MCF-7, and HepG2 cells, while compounds **34** and **40** had cytotoxic effects only on NCI-H460 and HepG2 cells	[21]
	*A. macrostemon*	**58, 71**	In vitro	-	Human neural carcinoma cells SF-268 and human large cell lung cancer cells NCI-H460	Compound **58** had cytotoxic effects on both SF-268 and NCI-H460 cells, while compound **71** had cytotoxic effects on SF-268 cells only	[96]
	*A. macrostemon*	AMB saponins	In vitro	It can reduce the mitochondrial membrane potential of HeLa cells, up-regulate Bax mRNA expression, down-regulate Bcl-2 mRNA expression and Bcl-2/Bax ratio, and enhance the activity of Caspase-9 and Caspase-3	Human cervical cancer HeLa cells	It can significantly reduce the mitochondrial membrane potential of HeLa cells, inhibit the proliferation of HeLa cells and promote their apoptosis	[98]
	*A. macrostemon*	**1**	In vitro and in vivo	Induces apoptosis by activating caspase activity, decreasing Bcl-2 expression, and inducing ROS production	A BALB/c nude mice xenograft model inoculated with human colon cancer cells SW-480	Significantly inhibits the proliferation of SW480 cells and induces apoptosis	[99]
	*A. chinense*	AMB saponins	In vitro and in vivo	By protecting the liver and spleen of mice, thus improving their immunity and inhibiting tumor cells	C57 BL/6 mice xenograft model inoculated with mice melanoma cells B16 and mice breast cancer cells 4T1	Inhibits the proliferation and induces apoptosis of B16 and 4T1 cells, and effectively protects the liver and spleen of mice	[100]
	*A. chinense*	*A. chinense* lectin	In vitro	Induced apoptosis in Hep-3B cells by upregulating the expression of caspase-3 and Bax	Human hepatocellular carcinoma cells Hep-3B	*A. chinense* lectin alters the morphological structure of Hep-3B and induces apoptosis	[101]
	*A. chinense*	**30, 84, 86, 88, 89**	In vitro	Induction of G_2_/M cell cycle arrest and apoptosis in HepG2 cells via a mitochondria-mediated pathway	Human hepatocellular carcinoma cell HepG2, human non-small cell lung cancer A549, human lung adenocarcinoma cell SPC-A-1, human gastric cancer cell MGC80-3, human breast cancer cell MDA-MB-231, human colon cancer cell SW620 and human nasopharyngeal cancer cell CNE-1	Inhibited all 7 types of cancer cells, but compound **84** only weakly inhibited HepG2 and CNE-1	[30]
Antibacterial effect	*A. macrostemon*	AMB aqueous extracts	In vitro	-	*Bacillus subtilis*, *Bacillus cereus*, *Staphylococcus aureus*, *Escherichia coli*, *Salmomella sp*, *Pseudomonas aeruginosa*	Inhibition ability in the order of *Staphylococcus aureus* > *Bacillus subtilis* > *Bacillus cereus* > *Escherichia coli* > *Pseudomonas aeruginosa* > *Salmomella sp*	[102]
	*A. chinense*	AMB extracts	In vitro	-	Candida albicans	Dimethyl trisulfide (**135**) 25.46% and methyl *cis*-1-propenyl disulfide (**115**) 14.69% higher content and better bacterial inhibitory effect	[169]
	*A. chinense*	AMB extracts	In vitro	Altered cell wall structure by disrupting the glycosidic bond of β-(1-3)-D glucan in the cell wall of Candida albicans	Candida albicans	It can inhibit the acidification of Candida albicans medium and cause the leakage of cellular OD_260nm_ substance, thus inhibiting its reproduction	[105]
	*A. macrostemon*	AMB fresh juice	In vitro	-	*Staphylococcus aureus*, *Escherichia coli*, *Bacillus subtilis*, *Proteus vulgaris*, *Enterobacter aerogenes*, *Alicrococcus tetragenus*, *Sarcina*, Brewer’s yeast, *Ranunculus repens*, *Aspergillus oryzae*, *Penicillium citrinum*, Trichoderma viride	The activity of antibacterial substances in the bulbs was higher than that of the above-ground parts, and in addition, the fresh juice of AMB had a significant inhibitory effect on both Gram-negative and positive bacteria, and on the spore germination of the test mycobacteria	[170]
	*A. macrostemon*	AMB 75% ethanol extracts	In vitro	-	*Staphylococcus aureus*, *Escherichia coli*, *Penicillium sp*, *Aspergillus niger*, *Saccharomyces cerevisiae*	The order of inhibition effect: *Escherichia coli* > *Staphylococcus aureus* > *Penicillium sp* > *Saccharomyces cerevisiae* > *Aspergillus niger*	[103]
	*A. chinense*	AMB saponins, AMB 30% and 60% ethanol extracts	In vitro	By reducing the utilization of glucose by bacteria, it affects the growth and reproduction of bacteria, reduces the activity of some key enzymes required for physiological metabolism, and thus inhibits the synthesis of related proteins.	*Staphylococcus aureus*, *Escherichia coli*, *Bacillus subtilis*, *Pseudomonas aeruginosa*, *Tritirachium album*, *Saccharomycete*	AMB saponins inhibited *Saccharomycete*, *Tritirachium album*, and *Staphylococcus* aureus, while the utilization of glucose by the above three bacteria treated with AMB saponins and AMB alcohol extracts was reduced, peroxidase activity was inhibited, and the total protein content of the bacteria decreased or even disappeared	[104]
Anti-asthmatic effect	*A. macrostemon*	AMB extracts	In vivo	-	Asthma model in guinea pigs by phosphate-histamine spray	AMB extract prolonged the latency period of asthma in guinea pigs, and the panting effect was enhanced with an increasing dose	[119]
	*A. macrostemon*	AMB saponins	In vitro	-	Histamine-induced constriction of isolated guinea pig tracheal lamellae model	Significantly relaxed histamine-induced spasm in isolated guinea pig bronchial smooth muscle	[120]
	*A. macrostemon*	AMB extracts	In vivo	Relieves chronic inflammation by suppressing the inflammatory response, which in turn relieves the spasticity of bronchial smooth muscle	Ultrasonic nebulization with 1% ovalbumin solution to produce an asthma model in guinea pigs	It can reduce the expression level of IL-6 and TXB_2_ and up-regulate the expression level of 6-Keto-PGF_1α_ in the serum of asthmatic guinea pigs, thus achieving the effect of calming asthma	[118]
Antioxidant effect	*A. macrostemon*	AMB extracts	In vivo	Increase the activity of antioxidant enzymes and promote the scavenging of free radicals	Rat model of liquor-induced oxidative stress	It can increase the activity of serum SOD and CAT in rats, has a protective effect on T lymphocytes, and significantly inhibits the formation of serum lipid peroxide	[138]
	*A. macrostemon*	AMB saponins	In vitro	-	-	It can effectively scavenge DPPH, O_2_^-^ and ·OH, and the antioxidant capacity of saponin components in AMB leaves is stronger than that of saponin components in bulbs	[139]
	*A. macrostemon*	AMB polysaccharides	In vitro	-	-	Sulfation modification of AMB polysaccharides by chlorosulfate-pyridine method can improve their in vitro antioxidant activity	[140]
	*A. macrostemon*	AMB polysaccharides	In vitro	-	-	Modification of AMB polysaccharides with α-amylase enhances their in vitro antioxidant activity	[141]
	*A. macrostemon*	AMB polysaccharides	In vitro	-	-	Relatively strong scavenging ability of AMB polysaccharide for ·OH	[142]
	*A. macrostemon*	Sulfur-containing compounds in AMB	In vitro and in vivo	Increase the activity of antioxidant enzymes and promote the scavenging of free radicals	Paraquat-methyl-^14^C induces oxidative stress in Cryptobacterium hidradenum	In vitro, the sulfur-containing compounds in AMB can effectively scavenge DPPH and ·OH and prevent the oxidation of Fe^2+^; in vivo, these sulfur-containing compounds can enhance the activity of SOD, GSH-Px, and CAT, thus promoting the scavenging of free radicals	[143]
Antidepressant effect	*A. macrostemon*	AMB aqueous extracts	In vivo	Promotes neurogenesis and BDNF release	Construction of a mice depression model using the behavioral desperation method of tail suspension and forced swimming	Ability to reduce immobility time and promote neurogenesis and BDNF expression levels in forced swim test and hanging tail test model mice	[151]
	*A. macrostemon*	AMB saponins	In vivo	Regulate the balance of the internal environment of depression model animals, such as hormone levels, at the same time, can significantly improve the pathological changes of related organs and tissues	Construction of a mice depression model using the behavioral desperation method of tail suspension and forced swimming; a mice depression model induced by intraperitoneal injection of reserpine; a rat model of chronic unpredictable depression by a 21-day chronic mild stimulation method	It can improve the tail suspension and swimming immobility time in mice with behavioral despair depression model, and also improve the body temperature decrease in mice with lisinopril depression model; in rats with chronic unpredictable depression model, it can also significantly improve the content of monoamine neurotransmitters 5-HT, NE, etc. in brain homogenate and serum corticosterone, adrenocorticotropic hormone levels, and improve the body immune function, and thymus, spleen, adrenal gland and hypothalamic nerve cell lesions	[150]
	*A. macrostemon*	AMB aqueous extracts	In vivo	-	Chronic stationary stress constructs a depression model in rats	Restores to normal levels several lysophosphatidylcholines and most medium and long chain acylcarnitines, phosphatidylcholines, and triglycerides that are abnormally altered in the plasma of depressed rats	[152]
Other pharmacological effects	*A. macrostemon*	AMB aqueous extracts	In vivo	-	Chemically and thermally induced pain mice model, NaNO_2_ poisoning and ISO-induced hypoxia mice model	Reduces the number and duration of writhing and foot-licking responses in model mice, and prolongs the duration of hypoxia tolerance in mice	[154]
	*A. macrostemon*	AMB extracts	In vivo	-	Non-specific and specific immune mice models were constructed by intravenous injection of ink and intraperitoneal injection of sheep red blood cells, respectively	It can increase the weight of the spleen and thymus, increase the carbon particle contouring index K and phagocytosis index α	[155]
	*A. macrostemon*	AMB aqueous extracts	In vivo	-	-	Significantly reduces the content of cytochrome P450 in mice, and has a significant inhibitory effect on hepatic drug enzymes	[157]
	*A. macrostemon*	AMB volatile oils, **113, 135**	In vivo	-	-	All of these have a strong killing effect on *Aedes albopictus* larvae	[158]
	*A. macrostemon*	AMB 30% ethanol extracts	In vivo	Regulation of bone formation and absorption	-	Increased expression of insulin-like growth factor-1 and bone morphogenetic protein-2, resulting in increased bone growth	[156]
	*A. macrostemon*	AMB aqueous extracts	In vitro and in vivo	Suppression of Nav1.7 channels	Chemically induced and thermally induced pain mice models	Reduces the number and duration of writhing and foot-licking responses in model mice and decreases the excitability of dorsal root ganglia by inhibiting Nav1.7 channels	[153]

## Data Availability

Data sharing not applicable to this article as no datasets were generated or analysed during the current study. Our manuscript does not produce new data; all available data are contained in the non published material.
